# Comparative analysis of avian hearts provides little evidence for variation among species with acquired endothermy

**DOI:** 10.1002/jmor.20952

**Published:** 2019-01-22

**Authors:** Jelle G. H. Kroneman, Jaeike W. Faber, Jacobine C.M. Schouten, Claudia F. Wolschrijn, Vincent M. Christoffels, Bjarke Jensen

**Affiliations:** ^1^ Department of Pathobiology, Anatomy and Physiology division, Faculty of Veterinary Medicine Utrecht University Utrecht The Netherlands; ^2^ Department of Medical Biology, Amsterdam Cardiovascular Sciences University of Amsterdam, Amsterdam UMC Meibergdreef 15, 1105AZ, Amsterdam The Netherlands

**Keywords:** anatomy, bird, evolution, heart

## Abstract

Mammals and birds acquired high performance hearts and endothermy during their independent evolution from amniotes with many sauropsid features. A literature review shows that the variation in atrial morphology is greater in mammals than in ectothermic sauropsids. We therefore hypothesized that the transition from ectothermy to endothermy was associated with greater variation in cardiac structure. We tested the hypothesis in 14 orders of birds by assessing the variation in 15 cardiac structures by macroscopic inspection and histology, with an emphasis on the atria as they have multiple features that lend themselves to quantification. We found bird hearts to have multiple features in common with ectothermic sauropsids (synapomorphies), such as the presence of three sinus horns. Convergent features were shared with crocodylians and mammals, such as the cranial offset of the left atrioventricular junction. Other convergent features, like the compact organization of the atrial walls, were shared with mammals only. Pacemaker myocardium, identified by Isl1 expression, was anatomically node‐like (Mallard), thickened (Chicken), or indistinct (Lesser redpoll, Jackdaw). Some features were distinctly avian, (autapomorphies) including the presence of a left atrial antechamber and the ventral merger of the left and right atrial auricles, which was found in some species of parrots and passerines. Most features, however, exhibited little variation. For instance, there were always three systemic veins and two pulmonary veins, whereas among mammals there are 2–3 and 1–7, respectively. Our findings suggest that the transition to high cardiac performance does not necessarily lead to a greater variation in cardiac structure.

## 1 INTRODUCTION

Mammals and birds evolved independently from reptile‐like ancestors as two vertebrate classes that are characterised by high metabolic rates and endothermy (Warren et al., 2008; Green et al., 2014; Tattersall, 2016). When the hearts of mammals and ectotherm sauropsids are compared, it is evident that mammalian hearts are remodeled by incorporation of systemic and pulmonary vein myocardium to the atria (Jensen, Boukens, Wang, Moorman, & Christoffels, 2014a; Carmona, Ariza, Cañete, & Muñoz‐Chápuli, 2018). The number of venous orifices to the left atrium can vary between one (dugongs) and seven (armadillos), and the myocardial sleeve of the veins may be extensive (mouse) or all but gone (harbour porpoise; Rowlatt, 1990; Mommersteeg et al., 2007). The number of venous orifices and the extent of myocardium even varies within the species (Nathan & Eliakim, 1966; Calkins et al., 2007, Rowlatt, 1990). In human, for example, the left atrium typically receives four pulmonary veins, but three or five veins are also frequently observed, and, although rare, two or six can also occur (Mansour et al., 2004). In contrast, in ectothermic sauropsids the pulmonary circulation connects to the left atrium by only a single orifice (Jensen, Moorman, Wang, 2014). These examples could suggest that the transition from ectothermy to endothermy, and the associated rise in cardiac pumping (Crossley et al., 2011), initiated greater variation in the morphology of the heart. We therefore hypothesized that hearts of birds exhibit a similar degree of variation to the hearts of mammals.

**Figure 1:**
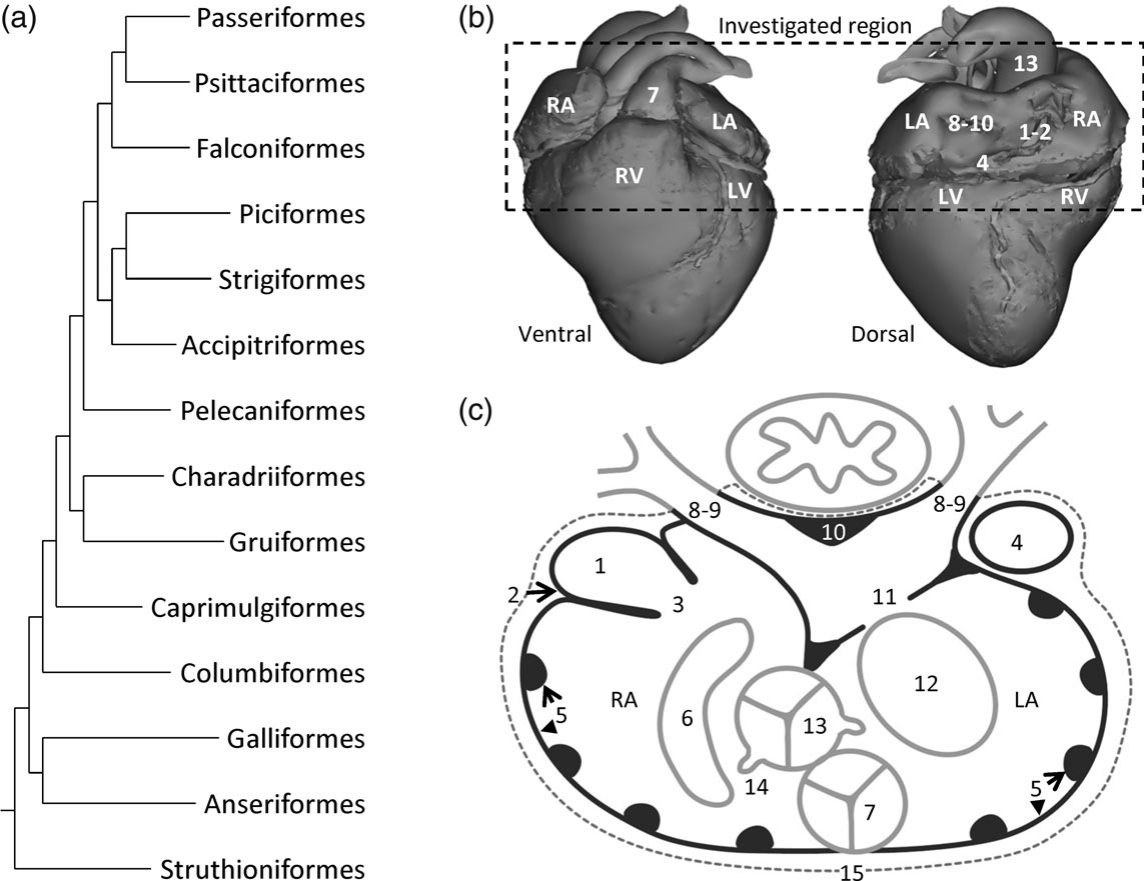
Phylogeny and scheme of the analysed structures of the avian hearts. (a) Phylogeny, on the basis of Jarvis et al. (2014), of the investigated orders. (b) In our investigations, we focused on the left and right atrium (LA and RA, respectively), the connecting veins, and the base of the left and right ventricle (LV and RV, respectively), and the arterial bases (see below for the number coding). In subsequent figure legends, we will refer to section numbers, where Section 1 represents the top horizontal line of the dashed box. (c) Schematic section in the transverse plane, which was the principle plane of sectioning, onto which we have collapsed all investigated structures: 1, myocardium in the sinus venosus; 2, the sinuatrial node; 3, sinuatrial valve and leaflets; 4, left sinus horn; 5, pectinate muscles of the atrial wall; 6, position of the right atrioventricular junction and muscularity of the valve; 7, position of the pulmonary artery and valve leaflets; 8, number of pulmonary veins; 9, the extent of pulmonary venous myocardium relative to the pericardium (dashed line); 10, dorsal ridge of the antechamber of the left atrium; 11, muscular shelf in the roof of the left atrium; 12, position of the left atrioventricular junction and muscularity of the valve; 13, position of the aorta and valve leaflets; 14, position of the orifices of the coronary arteries; 15, presence of ventral merger of the atrial walls

The extensive studies on the chicken heart, its development in particular, sharply contrasts the comparatively low number of anatomical studies on hearts of other birds (Hamburger & Hamilton, 1951; Van Mierop, 1967; Poelmann, Mikawa, & Gittenberger‐De Groot, 1998; Sedmera, Pexieder, Vuillemin, Thompson, & Anderson, 2000; Lincoln, Alfieri, & Yutzey, 2004; van den Berg et al., 2009; Bressan, Lui, Louie, & Mikawa, 2016). Literature on the venous‐atrial region in avian species is limited (Jensen et al., 2014a). In chicken, there is a myocardial sleeve surrounding the systemic and pulmonary veins that lie within the pericardial cavity (Endo, Kurohmaru, Nishida, & Hayashi, 1992; van den Hoff, Kruithof, Moorman, Markwald, & Wessels, 2001), but similar studies investigating this feature have, to the best of our knowledge, not been extended to other avian species. Older studies made macroscopic comparisons of multiple species (Gasch, 1888; Benninghoff, 1933) but a general paucity of images and quantifications makes it difficult to verify these findings, let alone make comparisons to other clades of vertebrates. For instance, the walls of the atria have been described as thin with thick bundles of muscle forming muscular arches (Whittow, 1999; Sedmera et al., 2000), but how this setting compares to other vertebrates remains difficult to assess. We therefore undertook a study to determine the variation in the morphology of the avian heart across species. We focused on the atria, the veins connecting to them, and the base of the ventricle in 14 orders of birds, assessing the 15 features schematized in Figure 1. The assessment of many of these features across vertebrates have previously been used to make phylogenetic inferences (Cook et al., 2017). We investigated one specimen *per* species in most instances and some of the variation we report may have arisen from individual variation, the state of tissue preparation, and experimental artefacts. Therefore, when we stress findings in any one particular specimen, it is primarily as evidence of variation to the general trends.

## 2 MATERIALS AND METHODS

### 2.1 Isolated hearts

Table 1 lists the species, the number of specimens per species, and the manner of investigation for the hearts used in this study. Figure 1a shows the relation of the investigated orders on the basis of the phylogeny of (Jarvis et al., 2014). Most hearts came from the Department of Pathobiology (Utrecht University). The age is not known for any specimen, except for the HH42 Chicken, but all animals were deemed to be of approximate adult size. The heart of the adult Chicken (*Gallus gallus*, Galliformes) came from an animal killed for human consumption, and the heart of a developing Chicken (Hamburger‐Hamilton stage 42) was isolated from an in‐house embryonated and incubated egg (Amsterdam UMC). One heart, isolated from an Ostrich specimen (*Struthio camelus*, Struthioniformes) was taken from a previously published model (Jensen et al., 2013). The heart of the Mallard (*Anas platyrhynchos*, Anseriformes) and Jackdaw (*Corvus monedula*, Passeriformes) were taken from culled animals. The heart of the Lesser redpoll (*Acanthis cabaret*, Passeriformes) was isolated from an animal that was found dead on a bike path (BJ).

**Table 1:** Species, number of specimens, and manner of investigation in this study. Macro, macroscopic inspection; Histology, series of histological sections stained with picro‐sirius red; Immnuo, fluorescent immumnohistochemistry

Species	Order	N	Macro	Histology	Immuno
Ostrich (*Struthio camelus*)	Struthioniformes	1	x		
Mallard (*Anas platyrhynchos*)	Anseriformes	1	x	x	PV, SAN
Chicken (*Gallus gallus*)	Galliformes	2	x	x	SAN
Collared dove (*Streptopelia decaocto*)	Columbiformes	1	x	x	PV
Common swift (*Apus apus*)	Caprimulgiformes	1	x	x	PV
Eurasian coot (*Fulica atra*)	Gruiformes	1	x	x	PV
Common snipe (*Gallinago gallinago*)	Charadriiformes	1	x	x	
Grey heron (*Ardea cinerea*)	Pelecaniformes	1	x		
Sparrowhawk (*Accipiter nisus*)	Accipitriformes	1	x	x	
Barn owl (*Tyto alba*)	Strigiformes	1	x	x	
Green woodpecker (*Picus viridis*)	Piciformes	1	x	x	PV
Common kestrel (*Falco tinnunculus*)	Falconiformes	1	x	x	PV
Budgerigar (*Melopsittacus undulatus*)	Psittaciformes	1	x	x	PV
Barn swallow (*Hirundo rustica*)	Passeriformes	1	x	x	PV
Hawfinch (*Coccothraustes coccothraustes*)	Passeriformes	1	x	x	
Western jackdaw (*Corvus monedula*)	Passeriformes	1	x	x	PV, SAN
Lesser redpoll (*Acanthis cabaret*)	Passeriformes	1	x	x	PV, SAN
Blackbird (*Turdus merula*)	Passeriformes	5	x	x	

Abbreviation: PV = immunohistochemical detection of pulmonary venous myocardium (cTnI); SAN = immunohistochemical detection of sinus nodal tissue (cTnI, SMA, Isl1); x = this manner of investigation was performed.

### 2.2 Tissue preservation

The adult Mallard, Jackdaw, Chicken HH42, and Lesser redpoll hearts were fixed in 4% PFA for 24 hr and then stored in 70% ethanol. The other samples came from hearts used in anatomy classes at the Department of Pathobiology: these had initially been frozen at −18 °C, thawed in water, fixed in 4% formaldehyde solution, and were afterwards stored in 1% formaldehyde solution. Before use in classes, the samples were rinsed for 48 hr with running tap water. This process had occurred an undetermined amount of times.

### 2.3 Histology and immunohistochemistry

The hearts that were investigated with histology (Table 1) were embedded in paraplast and cut in 10 μm transverse sections, except for the Collared dove which was cut in four‐chamber view. The principal staining was picro‐sirius red (muscle is stained orange, collagen red with a 2 min differentiation step in 0.01 mol L^−1^ HCl). The atrial region of each heart yielded several hundred to thousands of sections and we selected, at a fixed distance (dependent on the size of the heart), approximately 20 sections to represent the entire atrial region that ran from below the atrioventricular junction to the roof of the atria. Myocardium was principally assessed on picro‐sirius red stained sections, complemented with immunohistochemistry in a few sections per section series. Immunohistochemically, we detected myocardium with cTnT mouse antibodies (Thermo Fisher Scientific dilution 1:200, RRID:AB_11000742) or cTnI rabbit antibodies (Hytest 1:200, RRID:AB_154084) visualized by a fluorescently labeled secondary donkey–anti‐mouse antibody (Thermo Fisher Scientific, dilution 1:250, RRID:AB_141607) or donkey–anti‐rabbit antibody (Thermo Fisher Scientific, dilution 1:200, RRID:AB_2535792) respectively, coupled to Alexa 488. Arterial musculature (SMA) was detected with a mouse antibody to smooth muscle actin (Sigma‐Aldrich, dilution 1:250, RRID:AB_476701) visualized by a fluorescently labelled secondary donkey anti‐mouse antibody coupled to Alexa 555 (Thermo Fisher Scientific, dilution 1:250, RRID:AB_2536180). For the identification of the sinuatrial node, Isl1 goat antibodies (Neuromics, dilution 1:200, RRID:AB_2126323) visualized by a fluorescently labeled secondary donkey‐anti‐goat antibody coupled to Alexa 647 (Thermo Fisher Scientific, dilution 1:250, RRID:AB_2535864) were used. Nuclei were stained with DAPI (Sigma‐Aldrich, dilution 1:1000, D9542). *In situ* hybridization was performed as described previously (Jensen et al. 2017), with probes against cardiac troponin I (*cTnI*) and bone morphogenetic protein 2 (*Bmp2*; Somi, Buffing, Moorman, & van den Hoff, 2004).

### 2.4 Imaging

Imaging of the picro‐sirius red stained slides was done with a Leica DM5000 light microscope. For the larger sections, we merged multiple photos using the “Photomerge” function in Adobe Photoshop CS6, version 13.0.1. Several of the hearts contained large quantities of blood that stained in a color similar to that of the muscular walls. In many instances, to ensure clarity, these areas were masked with white to make the cardiac tissue stand out. If this was done, it is stated in the figure legend. For immunohistochemistry, slides were viewed and photographed with a Leica DM6000B fluorescent microscope. For the Barn swallow, Green woodpecker, and the Mallard, all analyzed structures were annotated in Amira software (version 6.0.0, FEI, SAS). To get volume estimates of each structure, the “Materials Statistics” tool in Amira was used (Supporting Information Figure 1). These three specimens were chosen because of the quality of the section series and because they span two orders of magnitude in heart and body size (approximately 10, 100, and 1,000 g in body weight).

### 2.5 Analysed structures

Figure 1 shows the 15 structures that were analysed: (1) Presence of myocardium in the sinus venosus, (2) location of the sinus node, (3) the sinuatrial valve and the number of leaflets it contains, (4) existence of a left sinus horn, (5) presence and size of pectinate muscles in the atrial wall, (6) anatomy and position of the right atrioventricular valve apparatus, (7) anatomy of the pulmonary arterial valve, (8) number of pulmonary veins entering the left atrium, (9) extent of pulmonary venous myocardium, (10) the dorsal myocardial ridge in the antechamber of the left atrium, (11) the muscular shelf in the left atrial roof, (12) anatomy and position of the left atrioventricular valve apparatus, (13) anatomy of the aortic valve, (14) origins of the coronary arteries, and (15) presence of ventral merger of the left and right atrial walls ventral to the pulmonary trunk.

All hearts were macroscopically inspected and were microscopically investigated with sections. Due to damage, not all structures could be assessed in all hearts. Supporting Information Table 1 lists for each heart which structures were investigated and in which manner.

## 3 RESULTS

In all investigated species, the heart was located dorsal to the sternum. The cardiac long‐axis was parallel to the sternum and the sternal carina, which ran parallel to the spine. The apex of the cardiac ventricles was the caudal‐most point of the heart (Figure 1b). All major arteries projected cranially from the ventricular base, and the atria were cranial to the ventricles (Figure 1b). The tissue that was close to the sternum was considered ventral, the tissue closest to the spine was considered dorsal.

### 3.1 Gross morphology

In all hearts, three large systemic veins enter the pericardial cavity, the right cranial vena cava, the left cranial vena cava, and the caudal vena cava. Their walls contain myocardium and are, therefore, considered to be derivatives of the sinus venosus, the chamber upstream of the right atrium in ectotherms (Figure 1c, structures 1 and 4). This is why the three vessels will be further referred to as the right sinus horn, left sinus horn, and caudal sinus horn. The opening of the sinus venosus into the right atrium always has a myocardial valve, comprising a left and a right leaflet (Figure 1c, structure 3). The luminal side of the wall of the right atrium has multiple pectinate muscles of varying sizes (Figure 1c, structure 5). The pectinate muscles converge cranially in a large muscular arch that spans the cranial part, or roof, of both atria from right to left, the so‐called transverse arch (it constitutes approximately a quarter of all atrial muscle, see table 2). At the medial‐caudal part of the right atrium, the entrance to the right ventricle is guarded by a large muscular flap valve (Figure 1c, structure 6), which, on the ventricular side, is made up of a continuation of ventricular myocardium, and on the atrial side of much thinner atrial muscle, with a thin layer of connective tissue in‐between the two layers.

**Table 2:** Proportions of atrial structures, estimated from areas in Amira. One specimen from each species was analysed

Structure\species	Mallard	Green woodpecker	Barn swallow
Tissue weight (g)	0,7	0.07	0,005
Sinus venosus	3%	5%	5%
Left sinus horn	5%	7%	7%
RA wall	20%	21%	29%
RA pectinate muscles	8%	11%	8%
Transverse arch	32%	20%	26%
Dorsal ridge	1%	4%	5%
LA shelf	2%	0,2%	0,5%
LA wall	17%	15%	13%
LA pectinate muscles	8%	15%	7%

Values are rounded to whole numbers, except for values less than 1.

Two pulmonary veins connect to the left atrium (Figure 1c, structure 8). They converge in an antechamber which, in turn, opens into the body of the left atrium (Figure 1c). In the dorsal wall of the antechamber, the myocardial walls of the pulmonary veins converge in a ridge of myocardium that is oriented parallel to the oesophagus (Figure 1c, structure 10). The antechamber is partly separated from the body of the left atrium by a muscular shelf hanging from the atrial roof (Figure 1c, structure 11). The walls of the antechamber are smooth, as opposed to the walls of the body of the left and right atrium, which are both trabeculated. A single atrial septum, without a *fossa ovalis*, separates the left and right atrium. This septum is continuous with the muscular shelf. The right atrium appears more voluminous than the left atrium, in part due to the somewhat left‐ward position of the atrial septum in combination with a greater caudo‐cranial height of the right atrial cavity. The entrance to the left ventricle is guarded by a valve with thin fibro‐membranous leaflets (Figure 1c, structure 12). From histology, we could not assert with certainty the number of leaflets of this valve. The outflow tract of both ventricles, the aorta and pulmonary artery, is partly embraced by the atria and are situated ventrally, the pulmonary artery being the most ventral of the two (Figure 1c, structures 7 and 13).

### 3.2 The sinus venosus

**Figure 2:**
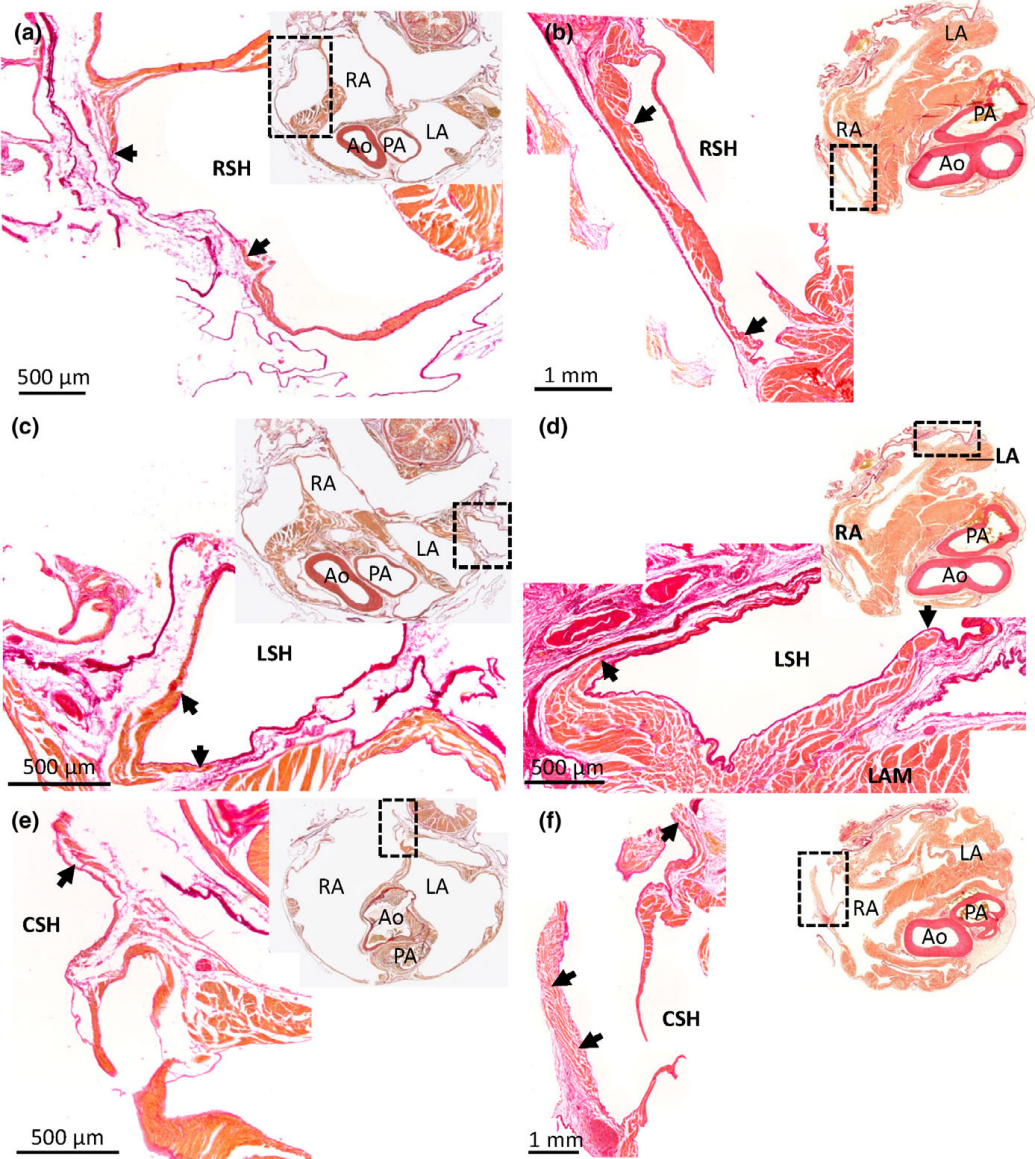
Myocardium in the sinus venosus of the Budgerigar (a, c, e) and the Mallard (b, d, f). Picro‐sirius red stained 10 μm histological sections in the transverse plane. Upper‐right corner inserts show the entire section, with the black boxes indicating the magnified part. The right sinus horn (RSH) (a, b), left sinus horn (LSH) (c, d), and caudal sinus horn (CSH) (e, f) contain myocardium (black arrows). The myocardial wall can be quite thick proximal to the atria, but tapers off distally. At the pericardial border, the vessel wall may be without myocardium. In the images of the Budgerigar, blood has been painted over with white for clarity. In the Budgerigar, sections 300 (cranial) to 850 (caudal) encompassed the atria and the sections shown are 425 (a), 400 (c), 600 (e). In the Mallard, sections 271 (cranial) to 1201 (caudal) encompassed the atria and the sections shown are 511 (b), 541 (d), 691 (f). Ao = aorta; LA = left atrium; LAM = left atrial muscle; PA = pulmonary artery; RA = right atrium

Myocardium was found in the left sinus horn, right sinus horn, and caudal sinus horn and it extended up to the proximity of the pericardium (Figure 2). In the birds analysed with Amira, the volume of the sinus venosus was compared to the volume of the right atrium (right atrial wall and right pectinate muscles) and was found to be approximately 12% (Table 2, Supporting Information Figure 1).

**Figure 3:**
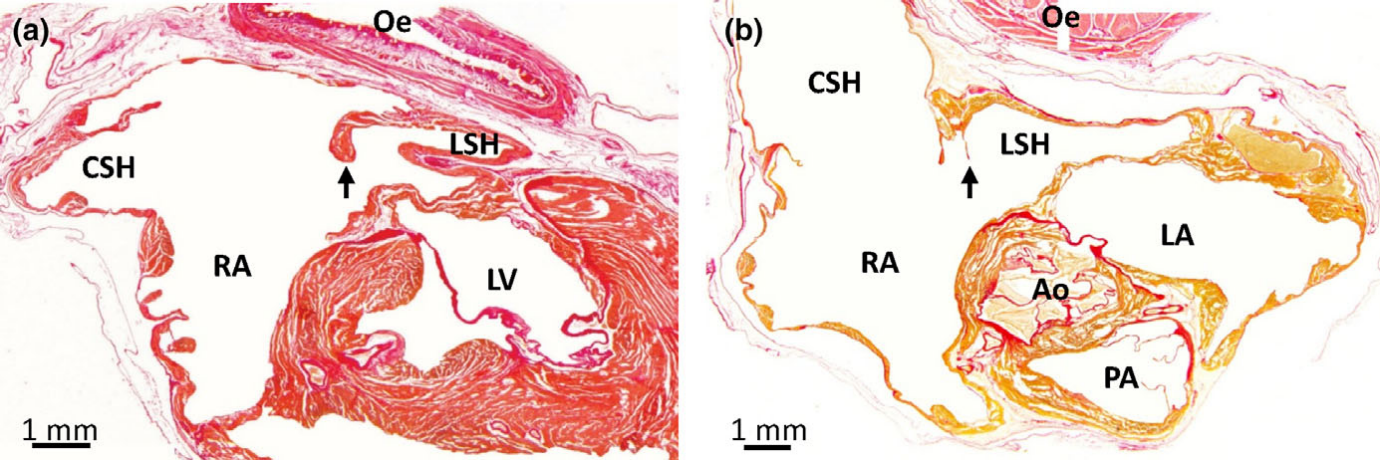
Leaflet of the valve of the left sinus horn in the Green woodpecker (a) and the Common kestrel (b). Birds generally have a prominent valve (black arrow) guarding the orifice of the left sinus horn (LSH), here exemplified by the Green woodpecker (section 1101, transverse plane, is shown where sections between 701 (cranial) and 1381 (caudal) encompassed the atria). It was found on sections representing 0.7 mm out of the total height of the atria of 6.8 mm. In the Common kestrel, the valve was a very thin membranous leaflet and it was found on sections representing 0.7 mm out of the total height of the atria of 6.6 mm (section 881, transverse plane, is shown where sections between 501 (cranial) and 1281 (caudal) encompassed the atria). Ao = aorta; CSH = caudal sinus horn; Oe = oesophagus; LA = left atrium; LV = left ventricle; LSH = left sinus horn; PA = pulmonary artery; RA = right atrium. In the images, blood has been painted over with white for clarity

The left sinus horn, proximal to its entry in to the right atrium, comprised of thick myocardial sleeve that fully surrounded the lumen (Figure 2). As the left sinus horn opened into the right atrium, some muscle protruded into the lumen as a myocardial leaflet (Figure 3). Such a leaflet was observed in all birds apart from the Common kestrel in which the leaflet was thin and membranous. Of the total volume of myocardium in the sinus venosus, the myocardium of the left sinus horn was approximately 60% (Table 2).

The entrance of the right sinus horn into the right atrium is separate from that of the caudal sinus horn and left sinus horn in most birds. Only in the Green woodpecker, the right sinus horn is part of a larger sinus venosus. The orifice of the right sinus horn is guarded by the sinuatrial valve, the left leaflet of which was best developed in this region of the right atrium. Cranially, the left leaflet has a thick muscular margin that merges with the transverse arch in the Common swift, Eurasian coot, Green woodpecker, and Budgerigar (Supporting Information Figure 2). Caudally, the right leaflet is more developed in the Mallard, Common kestrel, Jackdaw, and Barn swallow, whereas the left leaflet appears to be more developed in the Common swift, Eurasian coot and Budgerigar. The position of the orifice of the right sinus horn and caudal sinus horn varied between species. We measured these positions relative to a dorsal‐ventral axis that was established from the position of oesophagus, atrial septum, aorta, and pulmonary vein (Supporting Information Figure 3).

### 3.3 Sinuatrial node in bird hearts

**Figure 4:**
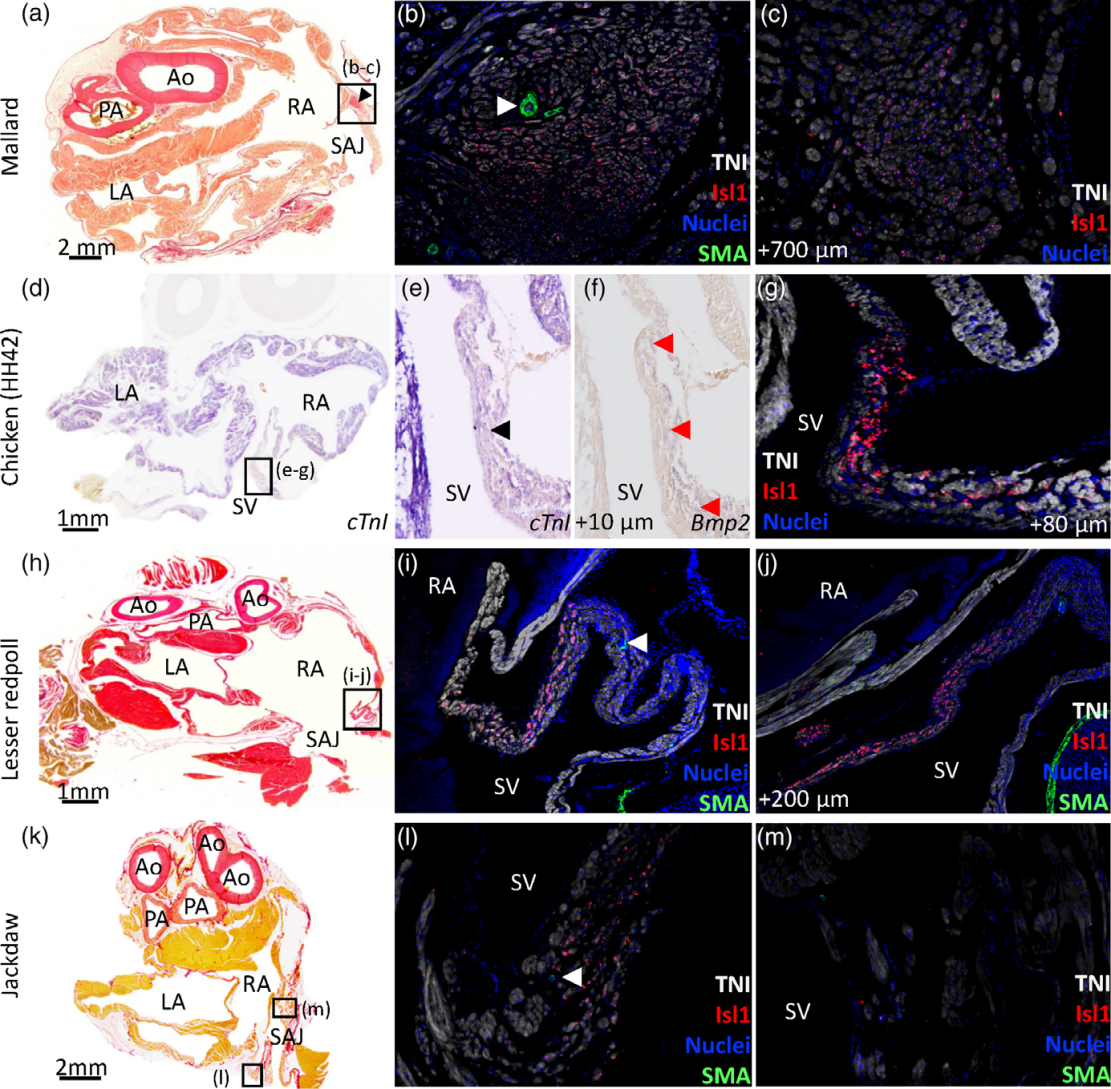
Sinuatrial node in Mallard (a–c), chicken HH42 (d–g), Lesser redpoll (h–j), and Jackdaw (k–m). All sections are in the transverse plane. The boxed areas in the left‐hand column images indicate the areas of the images of the middle and right‐hand columns. (a–c) In the Mallard, a nodal structure at the base of the right leaflet of the sinuatrial valve (black arrowhead in [a]) expressed Isl1 (b, c) and had a large coronary artery (white arrowhead in [b]). Sections 271 (cranial) to 1201 (caudal) encompassed the atria and the sections shown are 691 (a), 692 (b), 762 (c). (d–g) In the chicken HH42, the sinus venosus (SV) expressed the myocardial marker *cTnI* and this expression was relatively weak at the base of the right leaflet of the sinuatrial valve (black arrowhead in [e]). (f–g) The base of the right leaflet of the sinuatrial valve expressed *Bmp2* (red arrowheads in [f]) and Isl1 [g]). Sections 17 (cranial) to 297 (caudal) encompassed the atria and the sections shown are 80 (d‐e), 78 (f), 88 (g). The sections were from the mid‐height of the atria. (h–j) In the lesser redpoll, Isl1 was expressed in the base of the right leaflet of the sinuatrial valve. There was no nodal structure but the Isl1 expressing wall was thicker than the surrounding walls and contained a large coronary artery (white arrowhead in [i]). Sections 321 (cranial) to 621 (caudal) encompassed the atria and the sections shown are 401 (h), 402 (i), 422 (j). (k–m) In the Jackdaw an Isl1 positive area was seen in the left sinus venosus myocardium (l) in which a coronary artery was visible (white arrowhead in (l). At the base of the right sinuatrial leaflet no positive Isl1 cells could be seen (m). Sections 122 (cranial) to 332 (caudal) encompassed the atria and the sections shown are 190 (k) and 191 (l–m). Ao = aorta; LA = left atrium; PA = pulmonary artery; RA = right atrium; SAJ = sinuatrial junction. In the picro‐sirius red images blood has been painted over with white for clarity

Detection of Isl1, a marker of sinuatrial pacemaker cardiomyocytes, was successful in adult Mallard, Chicken HH42, Lesser redpoll, and Jackdaw, the best‐preserved specimens (Figure 4). In the Mallard, Isl1 detection was confined to a small oval‐shaped structure at the base of the right leaflet of the sinuatrial valve, resembling the sinus node of mammals (Figure 4a–c, Supporting Information Figure 4). At its most expansive part, the sinuatrial node cross‐section was approximately 700 μm wide and 900 μm long and it was detectable on eight sections each 300 μm apart, thus giving a total volume of approximately 1.5 mm^3^. A large coronary artery, identified by the expression of smooth muscle actin in the arterial wall, was found within the Isl1 positive domain, resembling the sinus nodal artery of mammals (Figure 4b). This domain was relatively rich in collagen compared to the surrounding myocardium of the sinus venosus and right atrium (Figure 4a). In Chicken, the presumptive pacemaker domain was anatomically less distinct than that in the Mallard. The base of the right leaflet of the sinuatrial valve expressed less *cTnI* than the surrounding muscle (Figure 4d,e), but uniquely expressed *Bmp2* (Figure 4f) and Isl1 (Figure 4g). In the Lesser redpoll, there was no anatomically identifiable node (Figure 4h). The base of the right leaflet of the sinuatrial valve was thicker than the surrounding walls, and this region expressed Isl1 (Figure 4i,j) and harbored a large coronary artery (Figure 4i). The Jackdaw had no anatomically distinct nodal structure (Figure 4k). An area on the caudal left side of the sinus venosus myocardium was Isl1 positive (Figure 4l). This area extended over an area of more than 600 μm in a caudo‐cranial direction. Isl1 was not detected at the base of the right sinuatrial valve (Figure 4m). In most specimens Isl1 could not be detected, but on the picro‐sirius red stained sections with reasonably good tissue presentation, a Mallard‐like sinus node was found in the Budgerigar only (Supporting Information Table 1). In the Common swift, Green woodpecker, and Common kestrel, there was no Mallard‐like sinus node, but rather thickened myocardium on the sinus‐side of the right leaflet of the sinuatrial valve that resembled the Isl1‐positive domain of the Lesser redpoll (Supportig Information Table 1). As these three species are phylogenetically positioned in‐between the Mallard and Budgerigar an obvious phylogenetic trend concerning the anatomy of the putative sinus node appears to be absent.

### 3.4 The right atrium

**Figure 5:**
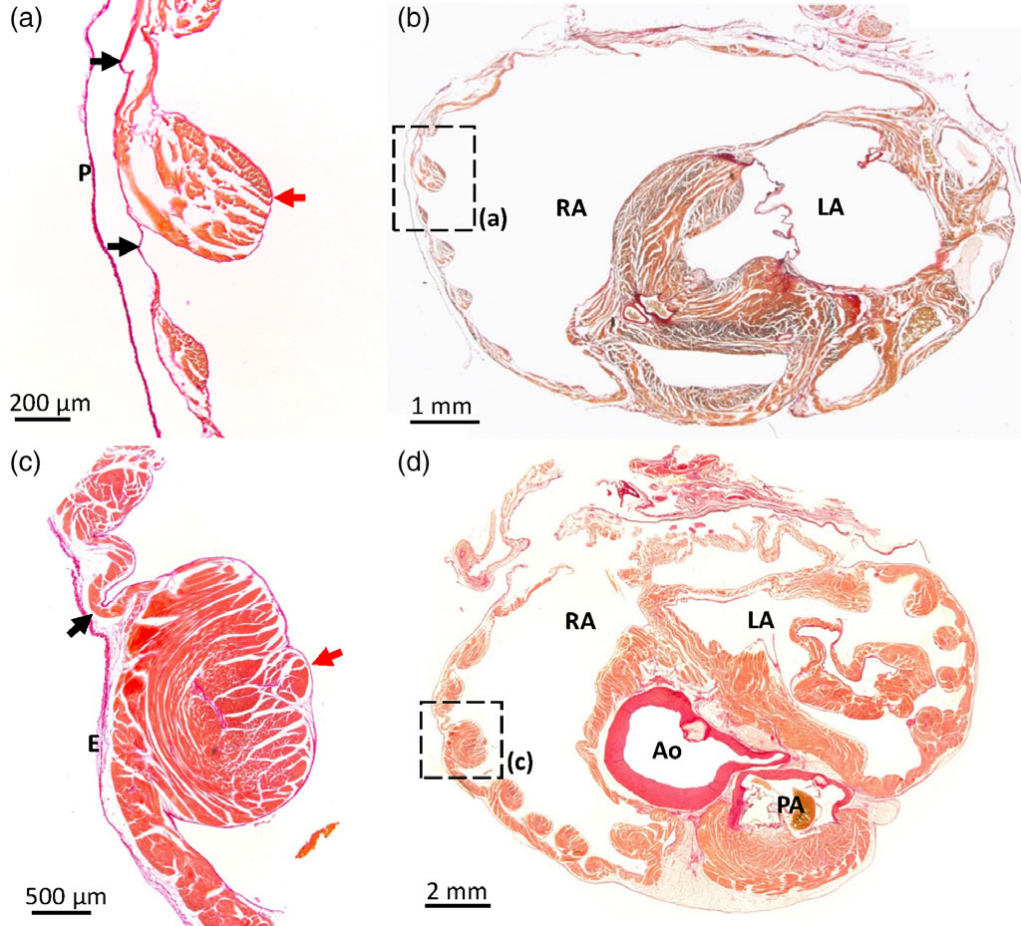
Atrial pectinate muscles in the Barn swallow and the Mallard. Picro‐sirius red stained sections in the transverse plane of Budgerigar (a, b; section 675 is shown where sections between 300 [cranial] to 850 [caudal] encompassed the atria) and Mallard (c, d; section 811 is shown where sections between 271 [cranial] and 1201 [caudal] encompassed the atria). The position of the pectinate muscles imaged in (a, c) are indicated by black squares in the overviews (b, d). Red arrows indicate pectinate muscles, black arrows point to the atrial wall in between the pectinate muscles. Note the several‐fold greater thickness of the pectinate muscles compared to the atrial wall. Ao = aorta; E = epicardium; LA = left atrium; P = pericardium; PA = pulmonary artery; RA = right atrium. In the images from the Budgerigar, blood has been painted over with white for clarity

In all bird hearts, extensive pectinate muscles were found in the right atrial wall. The smallest birds, Barn swallow and Lesser redpoll, had the fewest pectinate muscles, 4 and 5, respectively, whereas the much larger Mallard had approximately 18 pectinate muscles across both atria (we did not count the number of pectinate muscles in the Ostrich as it was not investigated by histology). Most pectinate muscles were nodular in cross section and much thicker than the atrial wall (Figure 5). In Budgerigar, a large trabecula was 0.56 mm in cross section while the atrial wall next to it was 0.021 mm thick, equal to a 27 fold difference in thickness. In Mallard, the trabecula was 8 times thicker, 1.79 versus 0.22 mm (Figure 5). The pectinate muscles came together in a large muscular transverse arch in the roof of both atria. In the birds analysed with Amira (Table 2), approximately half the volume of the atrial muscle was trabeculated (transverse arch, left atrial pectinate muscles, right atrial pectinate muscles, and dorsal ridge). The atrial septum was thin and a shallow depression akin to the foramen ovale of eutherian mammals was never observed.

### 3.5 Position of the atrioventricular orifices

**Figure 6:**
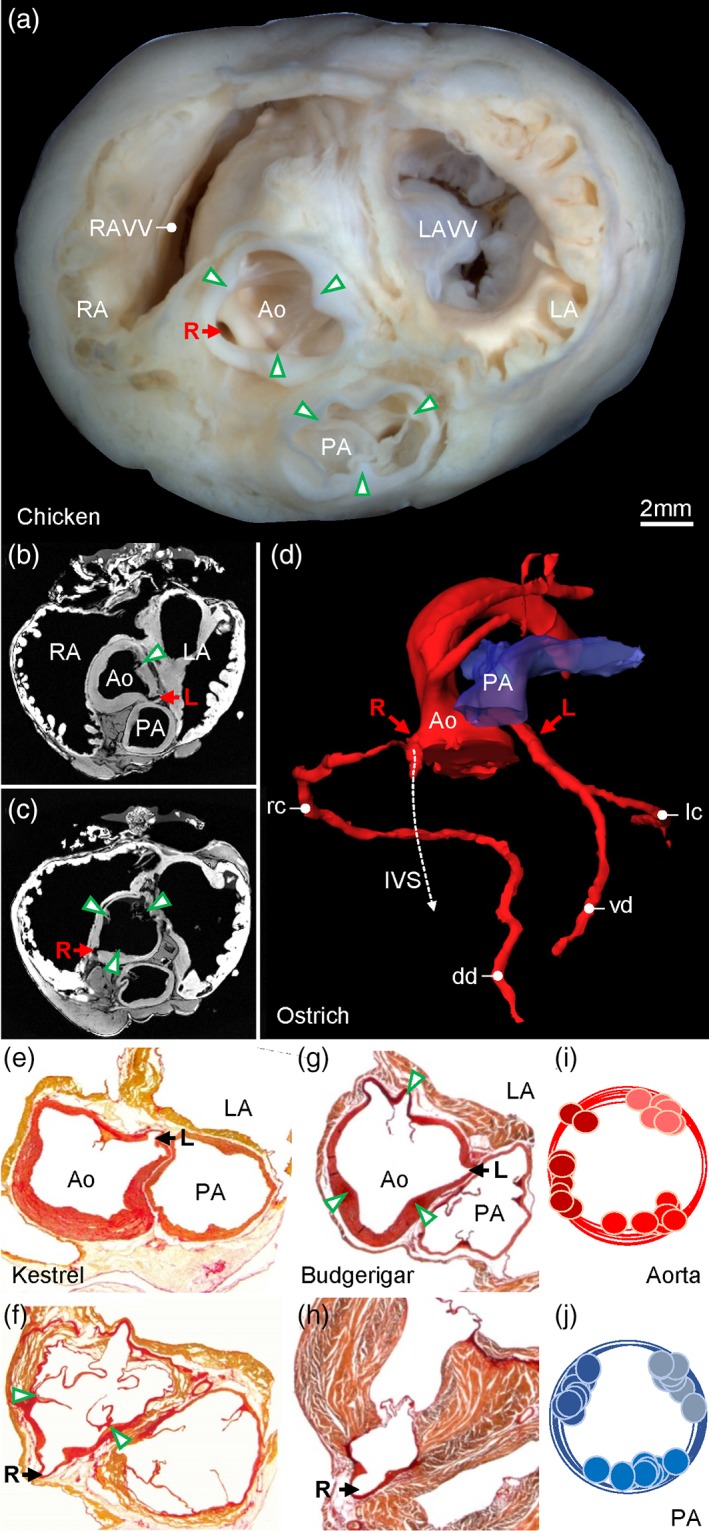
Major arteries. (a) Gross morphology of the ventricular base of the Chicken with indication of the valve commissures (green arrowheads) and the right coronary artery (R). (b, d) In the Ostrich, the left coronary artery (L) originates from the sinus of the ventral‐left leaflet of the aortic valve (b) and the right coronary artery (R) originates from the sinus of the ventral‐right leaflet of the aortic valve (c) (arrow heads indicate leaflet commissures). (d) The 3D reconstruction of the lumen of the major arteries shows the right coronary immediately splits into a branch that runs into the interventricular septum (IVS), next to the ventral merger of the right atrioventricular valve and the interventricular septum. The right coronary artery gives rise to the right circumflex artery (rc) leading to the dorsal descending artery (dd) in the interventricular sulcus. The left coronary artery splits into the ventral descending artery (vd) in the interventricular sulcus and the left circumflex artery (lc). The images of (b‐d) are derived and modified from the Ostrich 3D model published previously (Jensen et al., 2013). In the Common kestrel (e, f) and Budgerigar (g, h), the origin of the coronary arteries from the aorta was like in the Ostrich, and the intraseptal branch of the right coronary artery was always found to be located next to the ventral merger of the right atrioventricular valve with the interventricular septum. The cartoons (i, j) show the distribution of the commissures of the valve leaflets between all sectioned birds. Ao = aorta; LA = left atrium; LAVV = left atrioventricular valve; PA = pulmonary artery; RA = right atrium; RAVV = right atrioventricular valve

In transverse sections, the atrioventricular and arterial orifices were seen to be nestled together (Figure 6). The aortic valve was located almost in the center of the ventricular base. Relative to this position, the right atrioventricular junction was found dorsally to the right, the left atrioventricular junction was found dorsally to the left, and the pulmonary arterial valve was located ventrally (Figure 6). The right atrioventricular junction was configured as a C shape, with the medial margin formed by the interventricular septum and the parietal margin formed by the large muscular flap valve. Ventrally, this flap valve merged with the septal surface. The left atrioventricular junction (Figure 6) was always rounded and guarded by a valve of connective tissue that was anchored to the papillary muscles of the ventricle. When we inspected the histological sections from cranial to caudal, the leaflets of the left atrioventricular junction always appeared before the flap valve of the right atrioventricular junction (Supporting Information Table 2), showing there was always a caudo‐cranial offset between the two atrioventricular junctions (Supporting Information Figure 5). The Eurasian coot had the largest offset at 4.4 mm and the Common snipe had the smallest with 0.3 mm.

### 3.6 The pulmonary artery, aorta, and coronary arteries

All investigated pulmonary arterial valves had three leaflets of roughly equal size (Figure 6). The leaflets were anchored in two dorsal commissures and one ventral commissure (Figure 6j). The aortic valve was approximately of the same size as the pulmonary arterial valve and also exhibited three leaflets and commissures (Figure 6). The position of the pulmonary arterial valve showed little variation. In contrast, the position of the commissures of the aortic valve showed variation (Figure 6i) such that the dorsal right commissure could be dorsal right (e.g., Ostrich and Chicken, Figure 6a, c), lateral (e.g., Common kestrel, Figure 6f), or ventral right (e.g., Budgerigar, Figure 6g). The aorta always gave rise to two coronary arteries, with the right coronary artery originating from the sinus to the right of the ventral commissure and the left coronary artery originating from the sinus to the left of the ventral commissure (Figure 6). Immediately outside the sinus, the right coronary artery gave off a branch that descended into the ventricular septum (Figure 6d) where the flap valve of the right atrioventricular junction merged with the ventricular septum.

### 3.7 The left atrium

**Figure 7:**
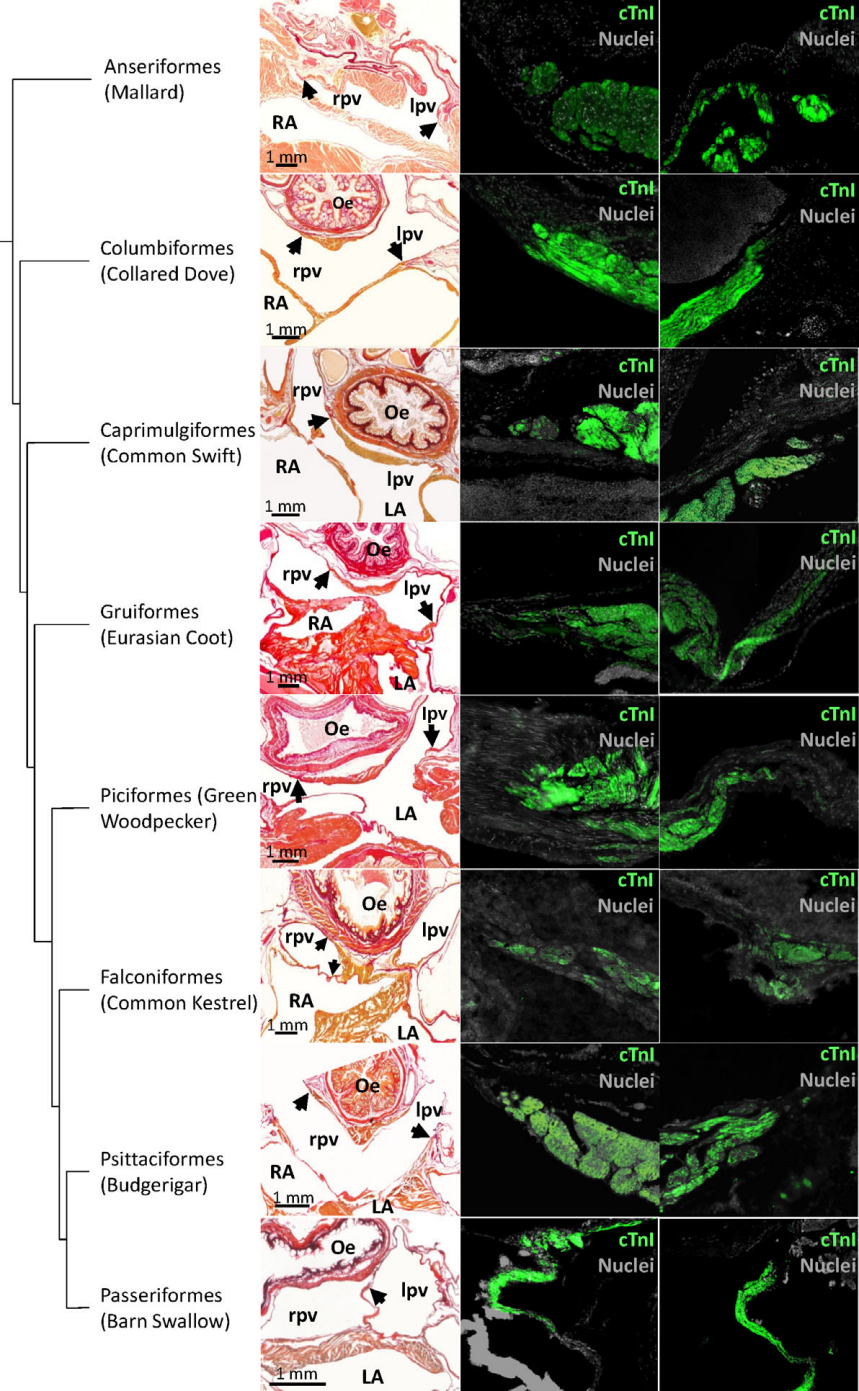
Pulmonary vein myocardium. In the first column picro‐sirius red stained sections. Black arrows point to the distal‐most extent of myocardium. The second and third column show immunohistochemical detection of cTnI at the cites indicated by the arrows. In the Common kestrel, only the right pulmonary vein is shown as the left pulmonary vein was damaged during sectioning. Oe = oesophagus; LA = left atrium; lpv = left pulmonary vein; RA = right atrium; rpv = right pulmonary vein. In all picro‐sirius red images, apart from the mallard, the blood has been painted over with white for clarity

In all species, only two pulmonary veins of equal size, a left and right, entered the pericardial cavity (Figure 7). A sleeve of myocardium was found around both pulmonary veins (Figure 7). With immunohistochemistry, it was confirmed that the myocardium always stopped in the vicinity of the pericardial reflection and never extended into the lungs, which were located further away from the border of the pericardial cavity (Figure 7, Supporting Information Figure 6). Beyond the myocardial sleeve, the thickness of the wall of the pulmonary vein decreased with 50% to 75%. The dorsal ridge, the myocardial sleeves of the pulmonary veins, together with the left atrial shelf formed an antechamber before the body of the left atrium. The prominence of the dorsal ridge was different between species (Table 2). The left atrial shelf formed the ventral boundary of the antechamber and was mostly muscular with some collagen. The free margin of the shelf pointed towards the atrioventricular junction. While the atrial shelf was found in all birds, its extent and thickness as compared to the surrounding wall varied between species (Table 2). The left atrial wall was thin, dominated by a few large pectinate muscles, similar to the right atrium.

### 3.8 Ventral merger of the atria

**Figure 8:**
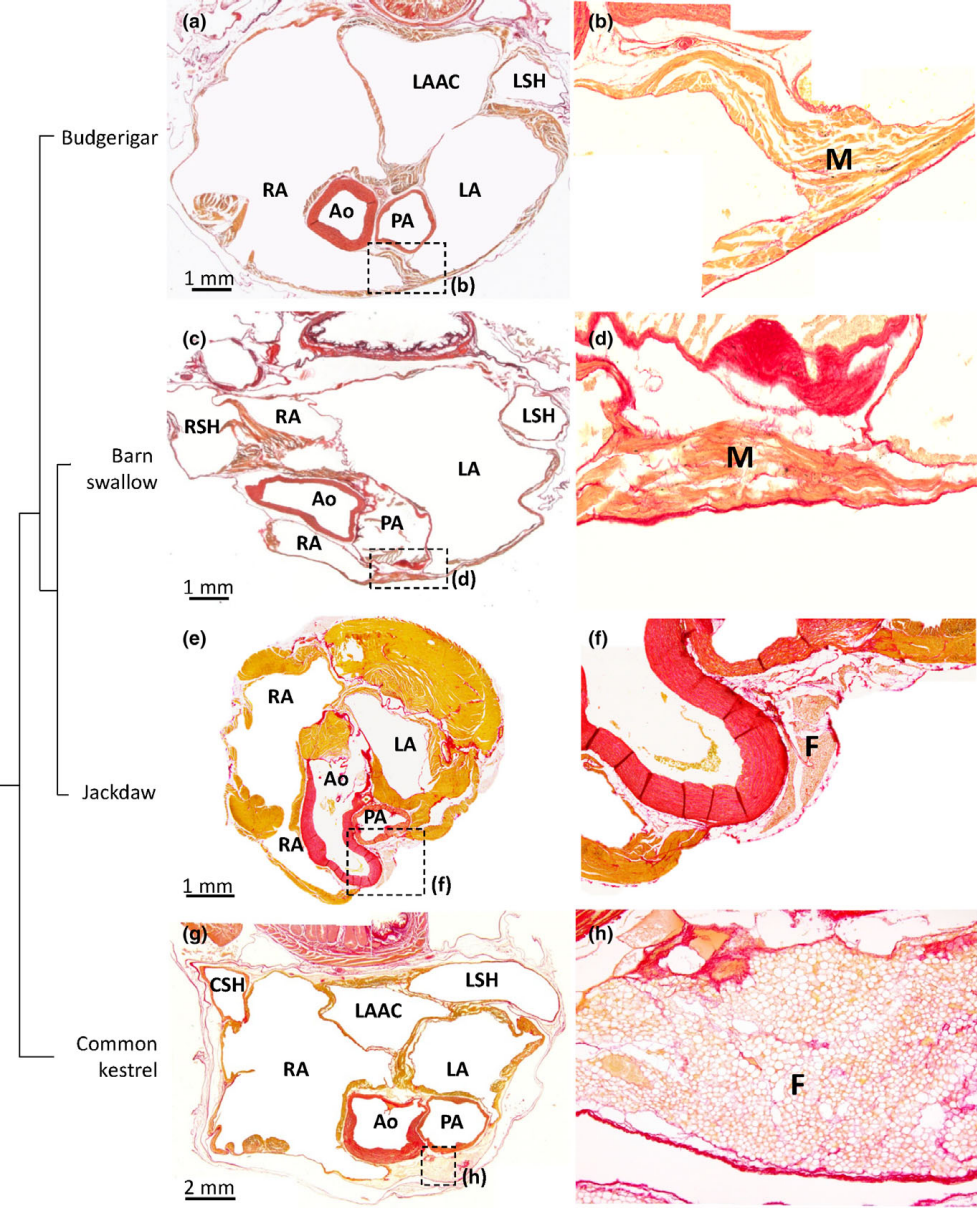
Ventral merger of the atrial walls. All sections are in the transverse plane. The boxed areas in the left‐hand column images, indicate the areas of the images of the right‐hand columns. (a, b) In the Budgerigar the atria were merged ventral to the pulmonary artery (PA), by connective tissue and myocardium (M) of the left (LA) and right (RA) atrium (section 475 [a, b] is shown where sections between 300 [cranial] to 850 [caudal] encompassed the atria). In the barn swallow (c, d), the myocardium of the left and right atrium is merged ventral to the pulmonary artery (section 210 [c, d] is shown where sections between 120 [cranial] to 540 [caudal] encompassed the atria). The Jackdaw (e, f; section 243 is shown where sections between 122 [cranial] and 332 [caudal] encompassed the atria) showed no ventral merger of the atria similar to the Common kestrel (g, h; section 721 is shown where sections between 501 [cranial] and 1281 [caudal] encompassed the atria). Ao = aorta; CSH = caudal sinus horn; F = fat; LAAC = left atrial antechamber; LSH = left sinus horn; RSH= right sinus horn. In all images, the blood has been painted over with white for clarity

In the Budgerigar (Psittaciformes) and the Barn swallow (Passeriformes), we found that the left and right atrium embraced the aorta and pulmonary artery such that their walls were fused ventrally to the pulmonary artery (Figure 8). The merger spanned approximately 1 mm from cranial to caudal. A similar merger was found in all five hearts of the Blackbird (Supporting Information Figure 7). In the section series of the Hawfinch heart (Passeriformes), only one section showed the walls of the left and right atrium to be merged, which appeared as a band of collagen. In the Lesser redpoll (Passeriformes), no merger was found, but the two atria were closely juxtaposed. This juxtaposition was not seen in the Jackdaw (Passeriformes). In other orders, the atria were further apart. Only in the Common kestrel (Falconiformes) the atria were close to each other, but still clearly separated by a pad of fatty tissue (Figure 8).

## 4 DISCUSSION

In the subsections below, we place our findings in an evolutionary context and summarize our study and literature by addressing synapomorphies (4.1), autapomorphies (4.2), convergent features (4.3), and variation (4.4). Concluding on these, the subsection (4.5) argues that the mammalian heart exhibits more variation than the avian heart. On this basis, we reject the hypothesis that the transition from ectothermy to endothermy and high cardiac performance is associated with greater variation in cardiac structure.

### 4.1 Gross morphological sauropsid synapomorphies of the bird heart

Birds are grouped in the archosaur clade, which also includes crocodylians (Green et al., 2014). Hearts of birds share several gross morphological features with crocodylians, and to a lesser extent with other ectothermic sauropsids. Our study extends the number of bird species that have been investigated and we show that birds, together with ectothermic sauropsids (Jensen et al., 2014b; Cook et al.*,* 2017), always have three sinus horns (Gasch, 1888; Benninghoff, 1933; Quiring, 1933; Adams, 1937; Rigdon & Frölich, 1970), and an atrial septum without a foramen ovale (Röse, 1890; Jensen, Wang, & Moorman, 2019). As in ectothermic sauropsids, the right atrium in birds is more voluminous than the left (Whittow, 1999; Jensen et al., 2014b). In birds and crocodylians, but not other sauropsids, the left atrioventricular valve comprises of membranes anchored in ventricular papillary muscles (Van Mierop &Kutsche, 1985; Lincoln et al., 2004; Alsafy, El‐Gendy, Enany, & Amine, 2009; Cook et al.*,* 2017). However, some features shared with crocodylians are more developed in birds. This includes the large muscular flap valve in the right atrioventricular junction (Gasch, 1888; Benninghoff, 1933; Bezuidenhout, 1983; Jensen et al., 2014b; Prosheva et al., 2015); the myocardial shelf between the antechamber and left atrial body, which is a meagre flap in crocodiles (Webb, 1979; Cook et al., 2017); and the offset between left and right atrioventricular junctions (Cook et al., 2017), which we confirm is much more pronounced in birds.

### 4.2 Gross morphological autapomorphies of the bird heart

The bird heart is readily distinct from the crocodylian heart and hearts of other sauropsids (Jensen et al., 2014b; Cook et al.*,* 2017) due to the opening of two pulmonary veins with myocardial sleeves into the left atrium. The pulmonary veins empty into a voluminous antechamber with a dorsal ridge of myocardium. A similar antechamber is not seen in crocodylians (Webb, 1979; Cook et al.*,* 2017). In birds, the myocardial shelf is found at some distance from the orifices of the pulmonary veins and the left atrioventricular junction. It could prevent regurgitation (Benninghoff, 1933), but given the distances to any orifice, it may guide blood toward the left ventricle instead. The myocardial shelf bears some resemblance to the membrane that divides the left atrium in the human congenital malformation of *cor triatriatum* (Bharucha et al., 2015). Benninghoff (1933) suggested that the myocardial shelf of birds develops from the left pulmonary ridge of the early embryonic atrium. A recent mouse model recapitulates *cor triatriatum* as seen in some patients, but it is not clear whether the morphology in that model is the outcome of aberrant development of the left pulmonary ridge (Muggenthaler et al., 2017).

Birds have one aorta with a tricuspid valve, ectothermic sauropsids have two aortae with bicuspid valves. In birds, the coronary arteries take their origins from the aorta much like in mammals, but, for example, the large intraseptal artery associated with the merger of the right atrioventricular valve to the ventricular septum is typically avian (Lindsay & Smith, 1965; Bezuidenhout, 1984; Kato, Narematsu, & Nakajima, 2018). The atrial walls of birds are dominated by a few large pectinate muscles coming together in the massive transverse arch in the atrial roof, whereas in ectothermic sauropsids the atrial walls consist of a thick meshwork of innumerous tiny trabeculae (Quiring, 1933; Rigdon & Fröhlich, 1970; Sedmera et al., 2000; Boukens et al., 2018). This atrial architecture in birds parallels the compact organization of their ventricular walls. Functionally, a compact wall architecture likely offers little impedance to blood flow and allows for fast electrical activation, thereby facilitating high heart rates (Boukens et al., 2018).

### 4.3 Convergent gross morphological features

The hearts of birds and mammals exhibit convergent features such as a single aorta, rather than the two aortae that ectothermic sauropsids have. Also, the aortic and pulmonary arterial valve are tricuspid, rather than bicuspid as in ectothermic reptiles (Benninghoff, 1933; Bartyzel, 2009a; Bartyzel, 2009b). The total number of arterial valve leaflets is thus the same between mammals, birds and ectothermic sauropsid (six leaflets), despite ectothermic sauropsids having a left aorta (two leaflets), a right aorta (two leaflets), and a pulmonary artery (two leaflets), reflecting evolutionary conserved morphogenetic processes (Poelmann et al., 2017). Like crocodylians and mammals (Cook et al.*,* 2017), birds have a full ventricular septum, and the atrioventricular junctions have an offset whereby the left junction is located cranial to that of the right (Adams, 1937). Our findings indicate that this offset is substantially greater in birds than in crocodylians and mammals. The pronounced offset in birds may reflect in part that the membranous septum of the ventricle completely myocardialises (Adams, 1937). In crocodylians this septum only exhibits slight myocardialization (Jensen et al., 2019) and mammals always have the membranous septum present in the adult heart (Rowlatt, 1990). We confirm the constant presence of a large left atrioventricular junction guarded by membranous leaflets anchored to papillary muscles, a feature crocodylians and birds share with mammals (Jensen et al., 2013; Cook et al.*,* 2017). The ventricular walls of mammalian and avian hearts are much less trabeculated than the ventricles of ectothermic vertebrates (Jensen et al.*,* 2016). Similarly, the atrial walls consist of a thick meshwork of trabeculae in ectotherms, whereas in birds there are some 10 pectinate muscles only, as had also been previously described in Chicken (Sedmera et al., 2000).

### 4.4 Variation between bird hearts

The ventral merger of the atria represents the clearest case for a feature that is not shared by all birds, and that may occur only within Passeriformes and Psittaciformes. This feature has not been described previously to the best of our knowledge, and is different from the myocardial bridges that embrace coronary vessels in mammals (Poláček, 1961; Van Nie & Vincent, 1989; Lee & Chen, 2015; Hostiuc, Negoi, Rusu, & Hostiuc, 2018). Given that the ventral merger was found in five out of five hearts of the Blackbird, it seems likely that it is a stable feature within a species even though it may exhibit much variation between species. In mammals, the left and right atrium can be identified by expression of Pitx2 and Bmp10, respectively (Kahr et al.*,* 2011), but the use of such markers to assess the relative contribution of the left and right atrium to the ventral merger has not yet been attempted in birds.

Isl1 could not be detected in most specimens, presumably due to the state of tissue preservation, but when we could detect it, the Isl1 expressing myocardium had substantial variation in morphology. One extreme was the Mallard, where Isl1 was confined to a nodal structure, not unlike mammals (Chiodi & Bartolomew, 1967). In contrast, in the Chicken, the Lesser redpoll, and the Jackdaw, Isl1 was detected in myocardium that, at best, could be distinguished as being slightly thicker than the surrounding walls of the sinus venosus and right atrium. This anatomically poorly defined setting has been recognized previously (Adams 1937; Chiodi & Bartolomew, 1967; Lamers, De Jong, De Groot, & Moorman, 1991) and strongly resembles the pacemaker region of ectothermic sauropsids (Jensen et al., 2017). Only in ectothermic sauropsids, the Isl1 expressing domain constitutes a ring immediately upstream of the sinuatrial junction (Adams, 1937; Jensen et al., 2017), whereas none of the birds studied here had such a ring. The mammalian sinuatrial node is located at the transition from the superior vena cava to the right atrium (Keith & Flack, 1907; Davies, 1942; Opthof, 1988; Boyett, Honjo, & Kodama, 2000; Chandler et al., 2009). Comparative anatomy placed the sinus node of birds at the base of the right sinuatrial leaflet (Davies, 1930; Adams 1937; Chiodi & Bartolomew, 1967; Kim & Yasuda, 1979; Lu, James, Yamamoto, & Terasaki, 1993) which has been confirmed in Chicken by electrophysiological and molecular studies (Moore, 1965; Bressan, Lui, Louie, & Mikawa, 2016). Extending those studies, we show in Chicken the colocalization of *Bmp2* and Isl1 which is also seen in the pacemaker tissue of Zebrafish (Tessadori et al., 2012) and *Anolis* lizards (Jensen et al., 2017). Note in the Lesser redpoll, however, that the Isl1‐positive domain extends caudal to the sinuatrial junction (Figure 4j) and that in the Jackdaw the Isl1‐positive domain was localized on the left side of the sinus venosus myocardium instead of the right. This indicates that not all birds, may have pacemaking originating from the base of the right sinuatrial valve leaflet as suggested on the basis of previous anatomical works (Chiodi & Bartolomew, 1967; Lamers, De Jong, De Groot, & Moorman, 1991). Also, we confirm the observation by Keith and Flack (1907) that the sinus node associates with a large coronary artery (Keith & Flack, 1907, Davies, 1930; Figure 4b,i,l). The Isl1‐detection of the presumed dominant pacemaker myocardium was successful in four specimens only, and additional studies are required to ascertain whether the variation we report here is the outcome of phylogenetic differences, which we consider likely, or the outcome of individual differences.

The state of development of the sinuatrial valve appeared to vary between specimens, and while this was a difficult feature to assess on the basis of histology, the thick valvar margin found in the Green woodpecker was unusual. The extent of myocardium around the pulmonary veins of all specimens was similar, but the extent of pulmonary venous myocardium may vary in the Chicken (Endo et al., 1992). In the Common kestrel, as in other birds of prey (Gasch, 1888), the left leaflet of the sinuatrial valve leaflet was thin and membranous. In the birds examined with Amira, we found the size of the sinus venosus relative to the right atrium to be 20% for Mallard and Barn swallow and 30% for Green woodpecker. In other clades, the mass of the sinus venosus is also a fraction of the atrial mass (20% for the Mako shark and 45% for the White sturgeon (Gregory et al., 2004)). It appears that the avian sinus venosus has enough muscle to aid atrial filling as it does in ectotherms, but it is not clear whether it actually does so (Jensen et al., 2017). Between specimens, the number of pectinate muscles in the atrial wall varied. This variation could reflect phylogeny, but size of the heart is likely another factor as we found the hearts of the smallest investigated birds, for example Barn swallow and Lesser redpoll, to have particularly few pectinate muscles. Generally, the position of atrioventricular junctions and arterial bases were fixed, but the aorta did exhibit some rotation in the transverse plane, although much less so than in mammals (Rowlatt, 1990).

### 4.5 The mammal heart is likely exceptional varied

Most of the investigated features of the bird heart exhibit less variation than the same features in mammal hearts. Birds always have three sinus horns, whereas mammals may have two, if the left sinus horn is regressed, or three. In mammals, the sinuatrial valve can be well‐developed and reptile‐like as in monotremes, well‐developed only around the inferior caval vein and coronary sinus, or almost completely regressed (Rowlatt, 1990; Jensen et al., 2014a). In contrast, both leaflets are always present in birds although their state of development varies as we show here and as has been noted before (Benninghoff, 1933; Adams, 1937).

The atrial septum of monotreme and marsupial mammals develops from the primary septum only. In eutherian mammals, the atrial septum is formed by the merger of the primary and secondary atrial septum, a process that is revealed in the adult heart by the presence of the fossa ovale (Röse, 1890; Runciman, Gannon, & Baudinette, 1995; Jensen, Wang, & Moorman, 2019). In contrast, the atrial septum of birds has no fossa ovale and it develops from the primary septum only (Jensen, Wang, Moorman, 2019).

The right atrioventricular junction in monotreme mammals is dominated by a large parietal flap valve, much like in birds and crocodylians, except for the fact that there is little, if any, myocardium in the monotreme valve (Adams, 1937; Dowd, 1969). In marsupial and eutherian mammals, the right atrioventricular valve consists of connective tissue only and typically has two or three leaflets anchored in papillary muscles (Rowlatt, 1990; Runciman, Baudinette, & Gannon, 1992). The right atrioventricular valve in all investigated birds was large, muscular, and without papillary muscles.

In monotreme mammals, the left atrium receives a single pulmonary vein like it does in ectothermic sauropsids. However, between genera of marsupial and eutherian mammals the number of pulmonary veins can vary between one and seven and myocardial sleeves may be absent, short, or extensive (Rowlatt, 1990). In contrast, two pulmonary veins connecting to the left atrium is a “konstant” feature of birds (Benninghoff, 1933). We confirm and extend on Benninghoff's observations and further show that the pulmonary veins have myocardial sleeves that extend up to the pericardial boundary. In mammals, the myocardial sleeves are often the cause of atrial fibrillation if they exhibit automaticity and re‐entry (Haïssaguerre et al., 1998; Chen et al., 1999; Tsuneoka, Koboyashi, Honda, Namekata, & Tanaka, 2012). It remains to be shown whether the pulmonary veins of birds are similarly arrhythmogenic.

In mammals, there is always a ventricular membranous septum which varies in size between genera, although it is always small compared to the muscular part of the septum, and which may be covered by a layer of myocardium (Rowlatt, 1990). Birds do form a membranous septum in development, but it undergoes myocardialisation and is subsequently lost. To the best of our knowledge, there are no descriptions of adult bird hearts with a persistent membranous septum. The large offset between left and right atrioventricular junctions in birds may in part result from growth of this myocardium of mesenchymal origin.

The relative position of the atrioventricular junctions and the base of the pulmonary artery and the aorta is not fixed across mammals (Rowlatt, 1990). The left and right atrioventricular junctions may be juxtaposed as seen in tree squirrels, or far apart, as seen in pygmy sperm whales. This, in turn, appears to reflect the process of aortic wedging, the extent to which the aorta has moved from its embryonic position on the right and toward the left ventricle (Cook et al.*,* 2017). In contrast, we found in all birds the same relative position of the atrioventricular junctions and the base of the pulmonary artery and the aorta. Only the commissures of the aortic valve appeared to exhibit some relative rotation, but such rotation appears to be much greater in mammals (Rowlatt, 1990).

The bird heart has several features that set it apart from that of ectothermic sauropsids but these features do not exhibit much variation. In contrast, the features that set mammals apart from their fellow amniotes, exhibit substantial variation. Therefore, our data do not support the hypothesis that the transition from ectothermy to endothermy associated with greater variation in cardiac morphology. While it is clear that there is a shared morphogenetic programme to the amniote heart (Keith & Flack, 1907; Olson, 2006; Jensen, Wang, Christoffels, & Moorman, 2013), the basis of the morphogenetic plasticity of mammals remains to be explored.

It is important to emphasize that some key features were not included in our study, such as the atrioventricular conduction axis, the extent of trabeculated ventricular muscle, or ventricular wall architecture. Between mammals, however, there is substantial variation in the extent of the atrioventricular conduction axis (Davies, 1942; Moorman, De Jong, Denyn, & Lamers, 1998) and the extent of trabeculated ventricular muscle (Rowlatt, 1990; Jensen et al., 2016). We do not know of data that suggests that the variation in these features among birds may be greater than it is in mammals. Also, even though heart weight varies substantially between birds (Nespolo et al., 2018) and, for example, Golden‐collared manakins have some 20% greater cardiac mass and left ventricular wall thickness than similar‐sized zebra finches (Barske et al., 2019) mammals also have a substantially varied, heart weight (Bishop, 1997; Seymour & Blaylock, 2000). For example, the heart weight of captive pronghorn antelopes is twice that of similarly sized goats (McKean & Walker, 1974).

## 5 CONCLUSION

We assessed 15 features of hearts of 14 orders of birds. Most features were surprisingly constant in appearance between orders, even those that were not shared with ectothermic sauropsids and thus considered autapomorphic. This suggests, in contrast to what was hypothesized, that the transition from ectothermy to endothermy, and the associated evolution of a high‐performance heart, does not necessarily lead to the extraordinary degree of variability in cardiac morphology that is observed in mammals. A greater number of features could have been assessed, but on the basis of literature we consider it unlikely the conclusion would change if additional features were included.

## AUTHOR CONTRIBUTIONS

JGHK and JWF performed experiments, analyzed data, and wrote the paper. JCMS procured most of the used material. CFW, VMC, and BJ designed the experiments, analyzed data, and wrote the paper.

## Supporting information


**Supplementary Figure 1 Amira example slides of Mallard and Green woodpecker**. (a) fully labeled mallard section in Amira. (b) picro‐sirius red section of the labeled section in a. (c) fully labeled Green woodpecker section in Amira. (d) picro‐sirius red section of the labeled section in d

**Supplementary Figure 2 Thick margin of the sinuatrial valve in the Green woodpecker**. The red arrow points to the thick margin of the valve which persists for 800 μm out of a total of 6,800 μm for the whole atria. Ao, aorta; Eso, esophagus; LA, left atrium; LSH, left sinus horn; PA, pulmonary artery; RA, right atrium

**Supplementary Figure 3 Variation in the position of the orifice of the sinus horns to the right atrium**. The colored ovals indicate the approximate size of the orifice to the size of the atria

**Supplementary Figure 4 Identification of the sinuatrial node in the Mallard**. Picro‐sirius red stained section the black square highlights a part of the sinuatrial junction(SAJ) while the black arrow points at the sinuatrial node(a). Cartoon of the sinus venosus with section lines marking the region that was examined(b). Immunohistochemistry sections of the area marked in panel a with the black square and the section lines in panel b. The TNI signal is marked white, Isl1 signal is marked red and nuclei signal blue this way co‐localization of Isl1 and nuclei turns purple(c‐f). Magnification of the area's marked with the white square in panel e and f respectively (e ‘, f‘). Ao, aorta; CSH, caudal sinus horn; Eso, esophagus; LA, left atrium; LSH, left sinus horn; PA, pulmonary artery; RA, right atrium; RSH, right sinus horn; V, ventricles

**Supplementary Figure 5 Offset in atrioventricular junctions in the Collared dove**. The left atrioventricular junction (LAVJ) is cranial to the right atrioventricular junction (RAVJ). LA, left atrium; LV, left ventricle; RA, right atrium; RV, right ventricle

**Supplementary Figure 6 Example examination of pulmonary vein myocardium in Collared dove**. Picro‐sirius red figures (a, d) of the section show general morphology of the atria and surrounding tissue. The black boxes in these images represent the locations of the immunohistochemistry images (b‐c, e‐f). Green marks the cTnI domain while gray marks nuclei. Eso, esophagus; LA, left atrium; LAAC, left atrial antechamber; LPV, left pulmonary vein; RA, right atrium; RPV, right pulmonary vein

**Supplementary Figure 7 Ventral merger of the atrial walls in the Common blackbird**. (a) Ventral face of Heart 1. The merger of the walls of the left atrium (LA) and the right atrium (RA) can be seen between the points of the two white arrows. The approximate positions of the transverse planes of sectioning of the histology of (b‐d) are indicated by dashed lines. (b‐d) Histological sections stained with picro‐sirius red, showing the merger of the atrial walls. (e‐h) Heart 2(e), 3(f), 4(g), and 5(h) also had merged atrial walls ventrally (between the points of the two white arrows)

**Supplementary Table 1** Overview of which structures were assessed in which species and by which manner of investigation.
**Supplementary Table 2**. The cranial offset of the left atrioventricular junction compared to the right atrioventricular junction
