# Supplementary figures and images for: Comparative analysis of avian hearts provides little evidence for variation among species with acquired endothermy
Source: J Morphol. 2019 Jan 22;280(3):395–410. doi: 10.1002/jmor.20952 (PMC6590421; doi:10.1002/jmor.20952)

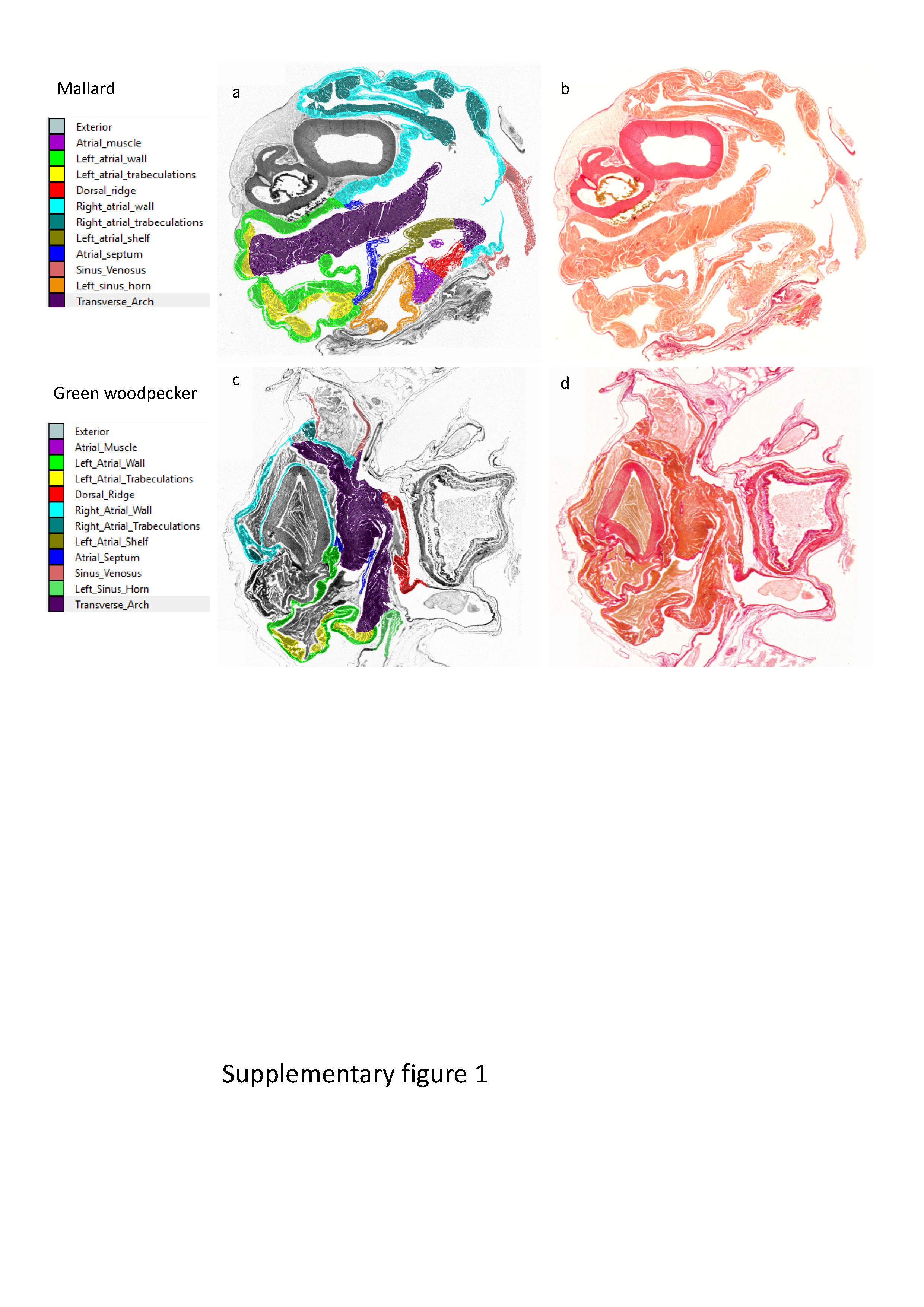

Supplement: Supplementary file 1 — Supplementary Figure 1 Amira example slides of Mallard and Green woodpecker. (a) fully labeled mallard section in Amira. (b) picro‐sirius red section of the labeled section in a. (c) fully labeled Green woodpecker section in Amira. (d) picro‐sirius red section of the labeled section in d [file JMOR-280-395-s001.tif]

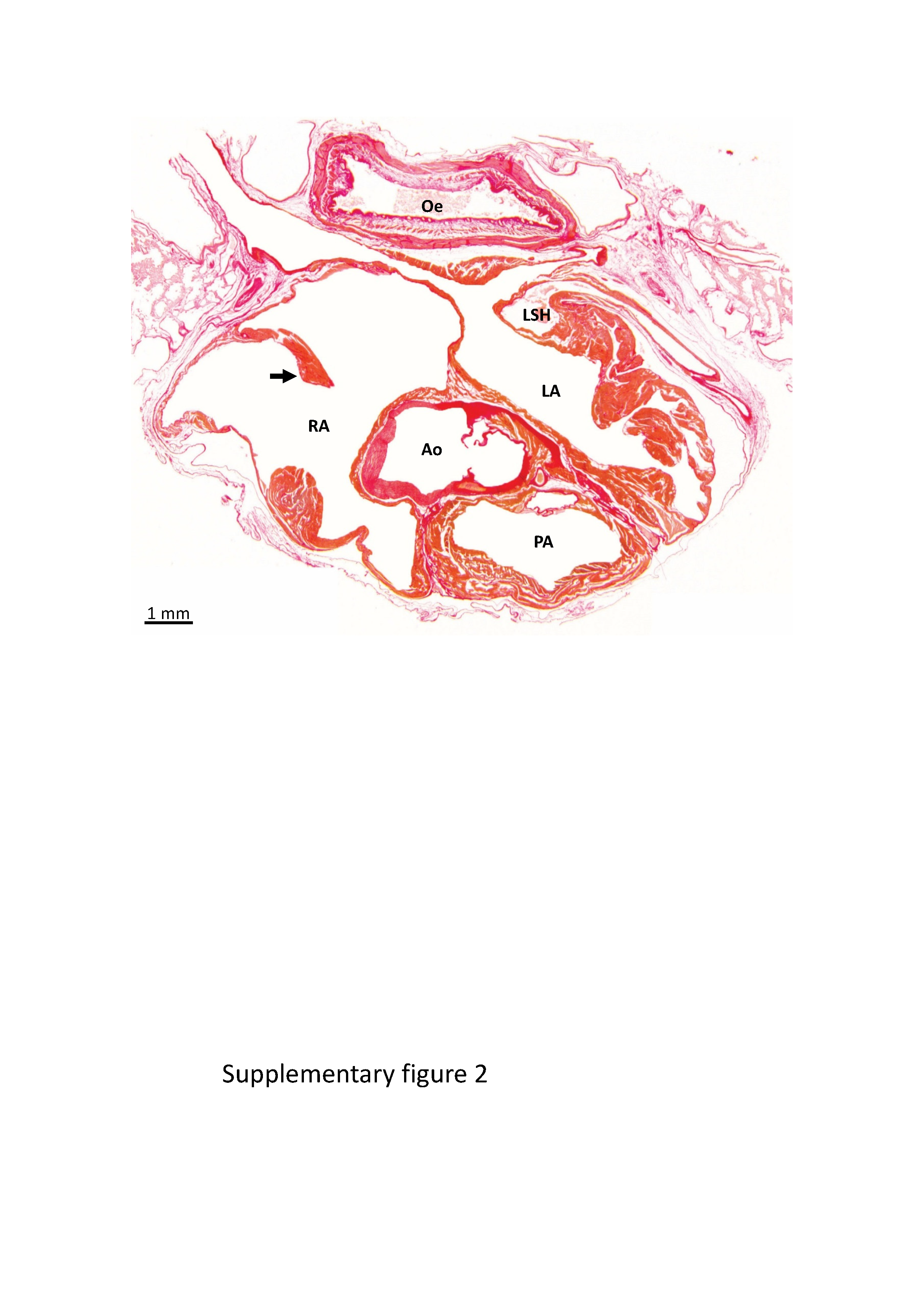

Supplement: Supplementary file 2 — Supplementary Figure 2 Thick margin of the sinuatrial valve in the Green woodpecker. The red arrow points to the thick margin of the valve which persists for 800 μm out of a total of 6,800 μm for the whole atria. Ao, aorta; Eso, esophagus; LA, left atrium; LSH, left sinus horn; PA, pulmonary artery; RA, right atrium [file JMOR-280-395-s002.tiff]

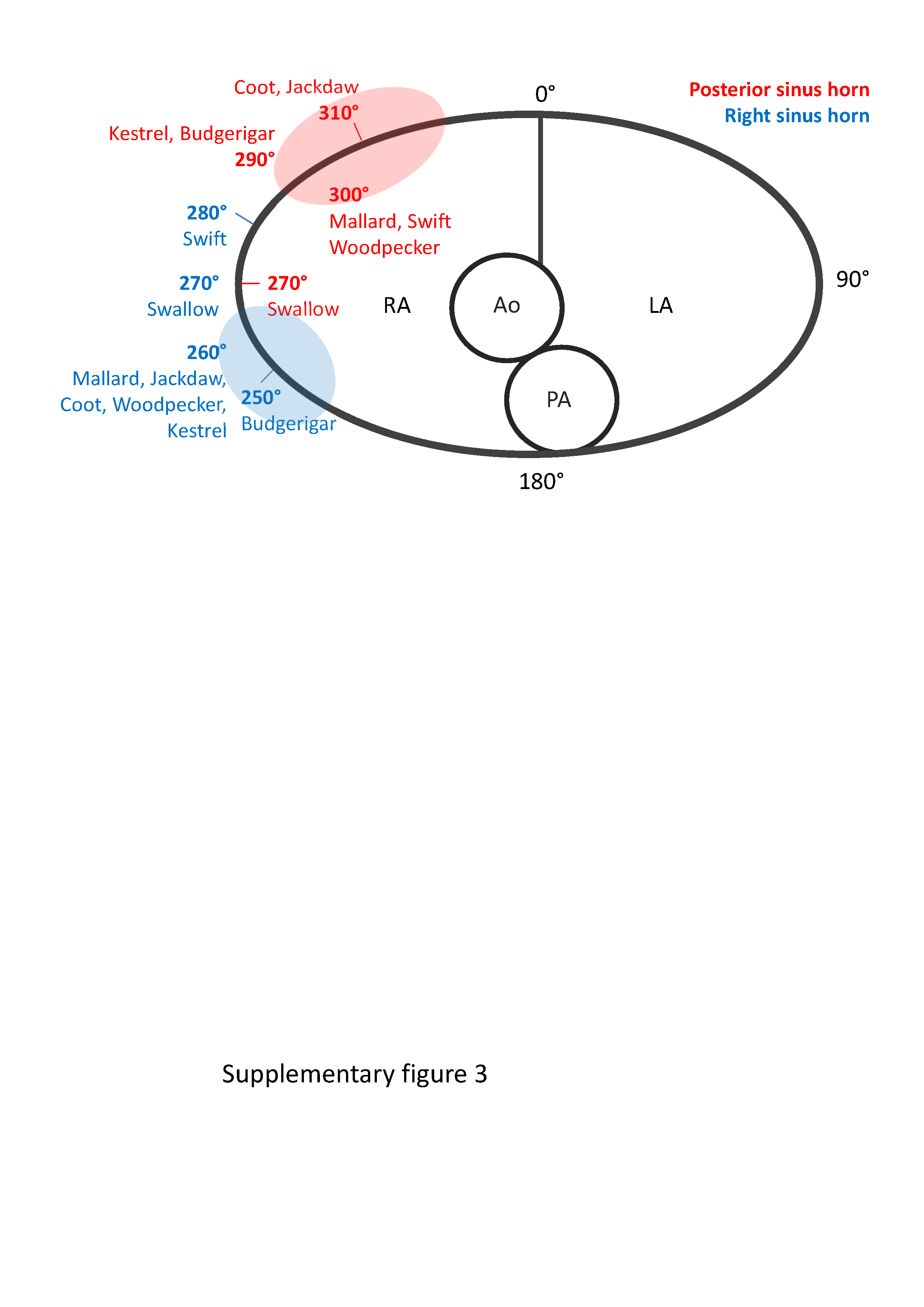

Supplement: Supplementary file 3 — Supplementary Figure 3 Variation in the position of the orifice of the sinus horns to the right atrium. The colored ovals indicate the approximate size of the orifice to the size of the atria [file JMOR-280-395-s003.tiff]

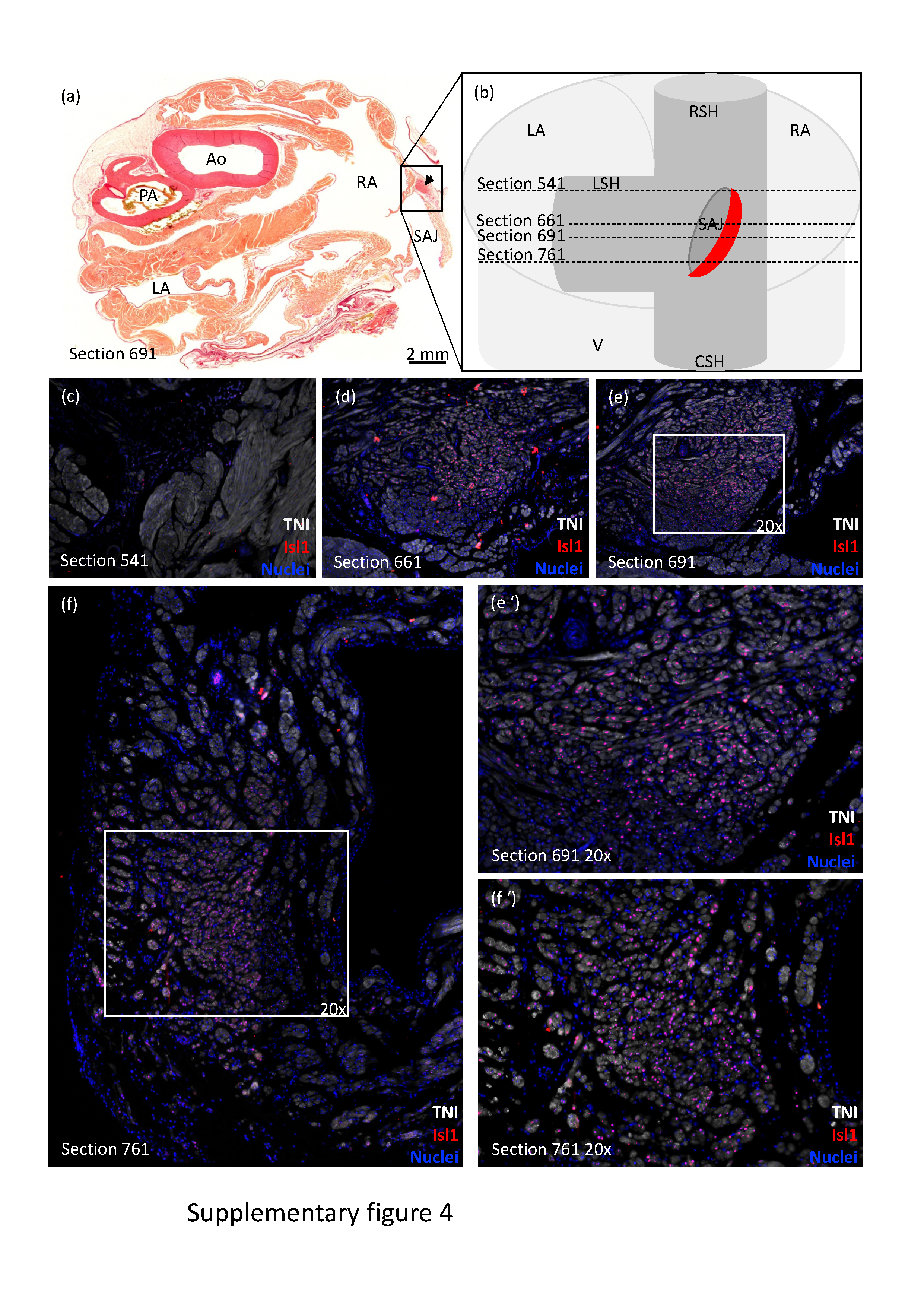

Supplement: Supplementary file 4 — Supplementary Figure 4 Identification of the sinuatrial node in the Mallard. Picro‐sirius red stained section the black square highlights a part of the sinuatrial junction(SAJ) while the black arrow points at the sinuatrial node(a). Cartoon of the sinus venosus with section lines marking the region that was examined(b). Immunohistochemistry sections of the area marked in panel a with the black square and the section lines in panel b. The TNI signal is marked white, Isl1 signal is marked red and nuclei signal blue this way co‐localization of Isl1 and nuclei turns purple(c‐f). Magnification of the area's marked with the white square in panel e and f respectively (e ‘, f‘). Ao, aorta; CSH, caudal sinus horn; Eso, esophagus; LA, left atrium; LSH, left sinus horn; PA, pulmonary artery; RA, right atrium; RSH, right sinus horn; V, ventricles [file JMOR-280-395-s004.tiff]

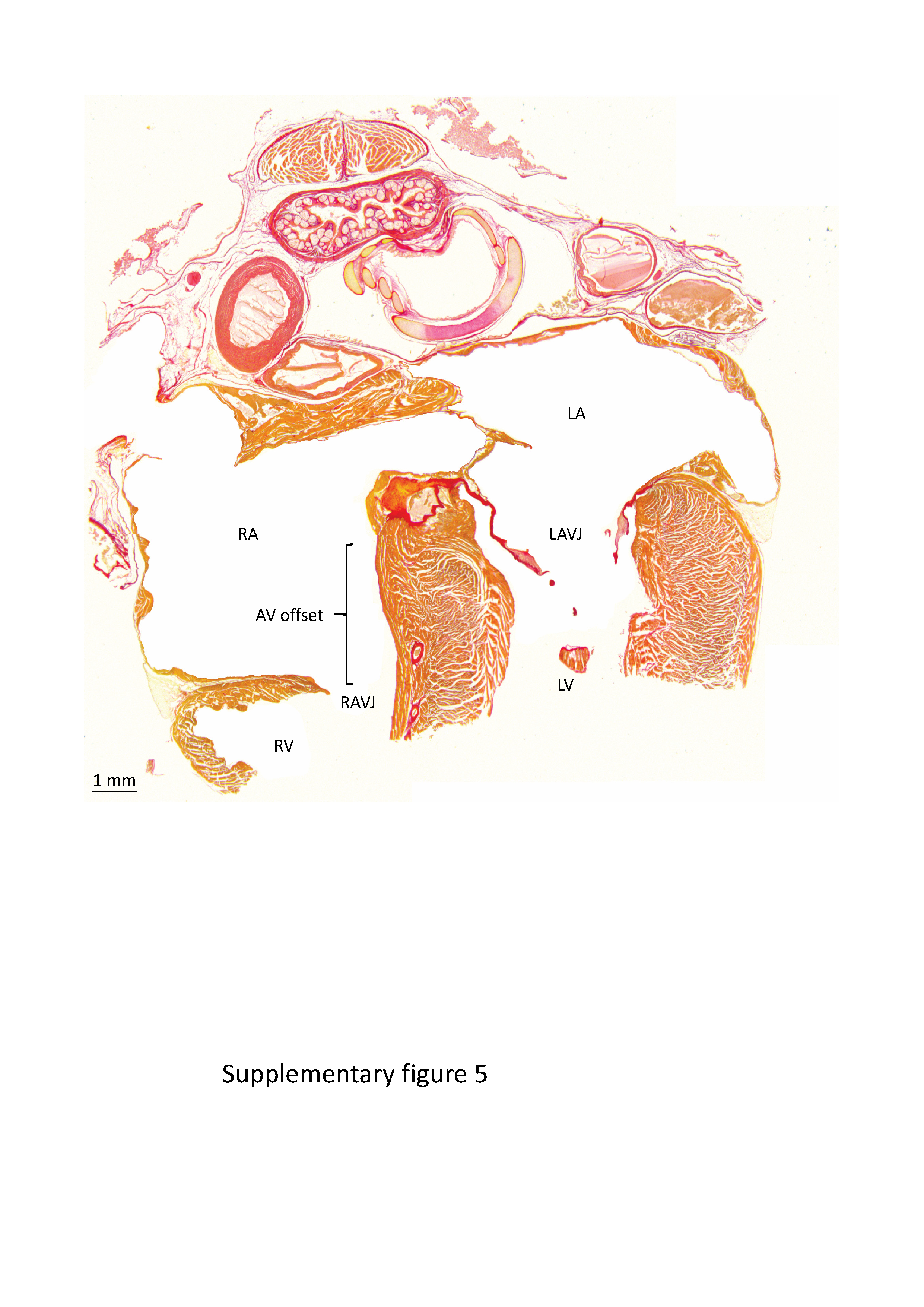

Supplement: Supplementary file 5 — Supplementary Figure 5 Offset in atrioventricular junctions in the Collared dove. The left atrioventricular junction (LAVJ) is cranial to the right atrioventricular junction (RAVJ). LA, left atrium; LV, left ventricle; RA, right atrium; RV, right ventricle [file JMOR-280-395-s005.tiff]

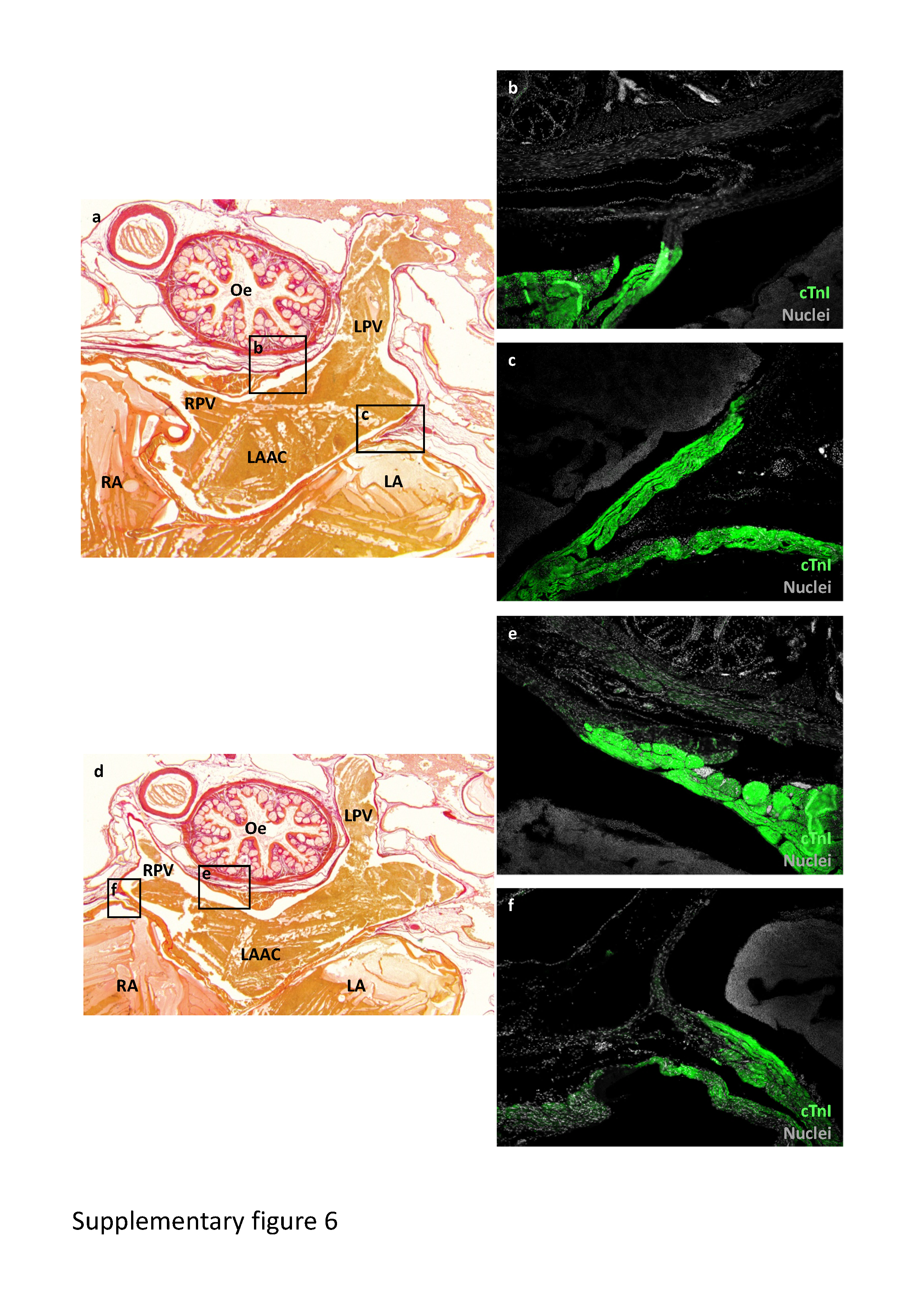

Supplement: Supplementary file 6 — Supplementary Figure 6 Example examination of pulmonary vein myocardium in Collared dove. Picro‐sirius red figures (a, d) of the section show general morphology of the atria and surrounding tissue. The black boxes in these images represent the locations of the immunohistochemistry images (b‐c, e‐f). Green marks the cTnI domain while gray marks nuclei. Eso, esophagus; LA, left atrium; LAAC, left atrial antechamber; LPV, left pulmonary vein; RA, right atrium; RPV, right pulmonary vein [file JMOR-280-395-s006.tiff]

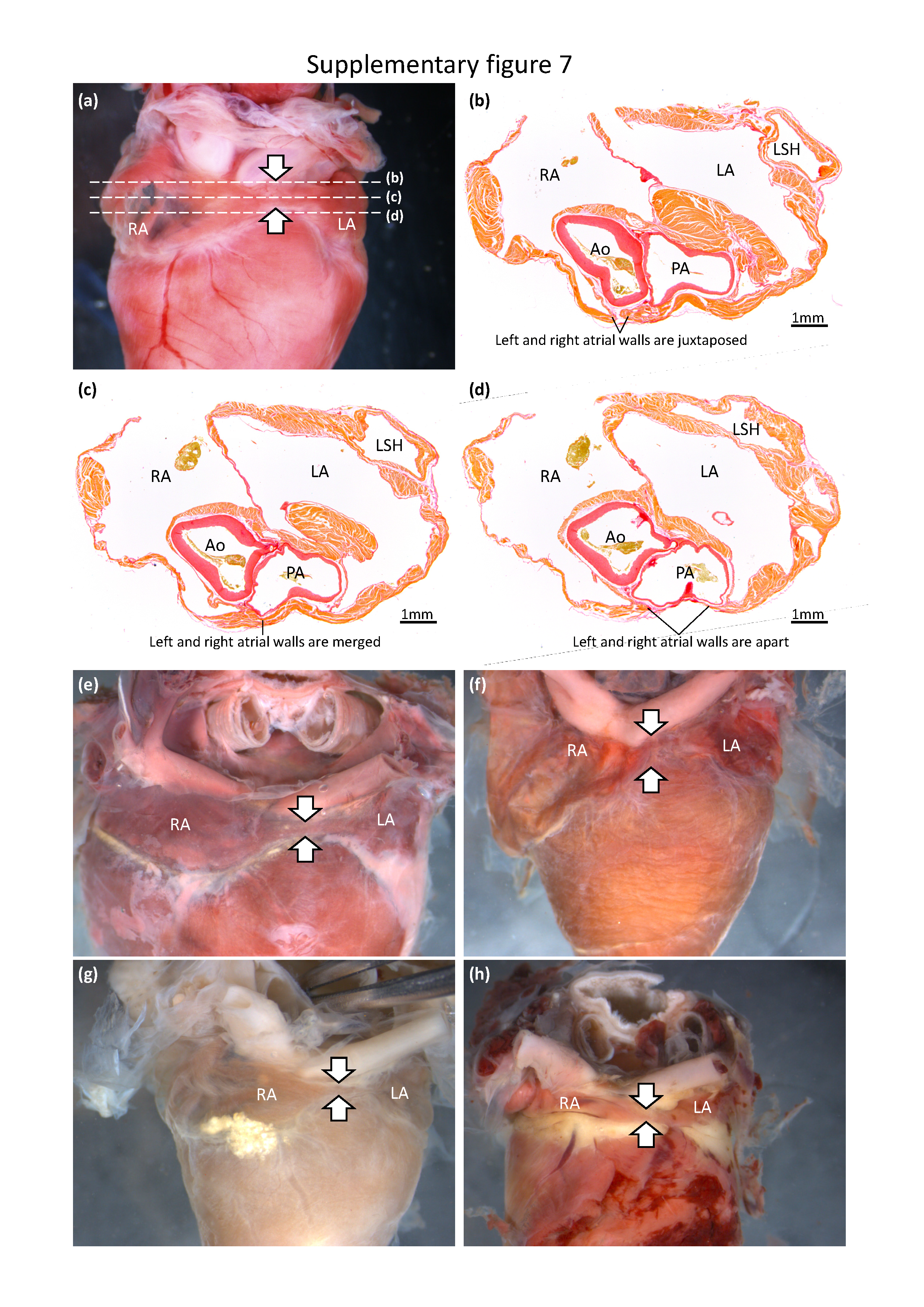

Supplement: Supplementary file 7 — Supplementary Figure 7 Ventral merger of the atrial walls in the Common blackbird. (a) Ventral face of Heart 1. The merger of the walls of the left atrium (LA) and the right atrium (RA) can be seen between the points of the two white arrows. The approximate positions of the transverse planes of sectioning of the histology of (b‐d) are indicated by dashed lines. (b‐d) Histological sections stained with picro‐sirius red, showing the merger of the atrial walls. (e‐h) Heart 2(e), 3(f), 4(g), and 5(h) also had merged atrial walls ventrally (between the points of the two white arrows) [file JMOR-280-395-s007.tiff]
