# Supplementary material for: Comparative analysis of avian hearts provides little evidence for variation among species with acquired endothermy
Source: J Morphol. 2019 Jan 22;280(3):395–410. doi: 10.1002/jmor.20952 (PMC6590421; doi:10.1002/jmor.20952)
Supplement: Supplementary file 8 — Supplementary Table 1 Overview of which structures were assessed in which species and by which manner of investigation. Supplementary Table 2. The cranial offset of the left atrioventricular junction compared to the right atrioventricular junction [file JMOR-280-395-s008.docx]

**Supplementary Table 1**. Overview of which structures were assessed in which species and by which manner of investigation.

| Species\Structure | 1 | 2h | 2i | 3 | 4 | 5 | 6 | 7 | 8 | 9 | 10 | 11 | 12 | 13 | 14 | 15 |
| --- | --- | --- | --- | --- | --- | --- | --- | --- | --- | --- | --- | --- | --- | --- | --- | --- |
| Ostrich | - | - | - | M+MRI | M+MRI | M+MRI | M+MRI | M+MRI | M+MRI | - | M+MRI | M+MRI | M+MRI | M+MRI | M+MRI | M+MRI |
| Mallard | Yes | Yes | Yes (on right) | Yes | Yes | Yes | Yes | Yes | Yes | Yes | Yes | Yes | Yes | Yes | Yes | Yes |
| Jackdaw | Yes | Yes | Yes | Yes | Yes | Yes | Yes | Yes | Yes | Yes | Yes | Yes | Yes | Yes | Yes | Yes |
| Chicken | Yes | Yes | SA thickening (on right) | M | M | - | M | M | M | - | - | - | M | M | M | M |
| Lesser Redpoll | Yes | Yes | SA thickening (on right) | Yes | Yes | Yes | Yes | Yes | Yes | Yes | Yes | Yes | Yes | Yes | Yes | Yes |
| Collared Dove | Yes | - | - | Yes | Yes | Yes | - | - | Yes | Yes | Yes | Yes | - | - | - | Yes |
| Common Swift | Yes | - | SA thickening (on right) | Yes | Yes | Yes | Yes | Yes | Yes | Yes | Yes | Yes | Yes | Yes | Yes | Yes |
| Eurasian Coot | Yes | - | - | Yes | Yes | Yes | Yes | Yes | Yes | Yes | Yes | Yes | Yes | Yes | Yes | Yes |
| Common Snipe | Yes | - | - | Yes | Yes | Yes | D | D | Yes | Yes | Yes | Yes | D | D | D | Yes |
| Sparrowhawk | LSH | - | - | D | Yes | Yes | Yes | Yes | D | D | D | D | Yes | Yes | Yes | Yes |
| Barn Owl | D | D | - | D | D | D | D | D | D | D | D | D | D | D | D | D |
| Green Woodpecker | Yes | - | SA thickening (on right) | Yes | Yes | Yes | Yes | Yes | Yes | Yes | Yes | Yes | Yes | Yes | Yes | Yes |
| Common Kestrel | Yes | - | SA thickening (on right) | Yes | Yes | Yes | Yes | Yes | Yes | Yes | Yes | Yes | Yes | Yes | Yes | Yes |
| Budgerigar | Yes | - | Yes | Yes | Yes | Yes | Yes | Yes | Yes | Yes | Yes | Yes | Yes | Yes | Yes | Yes |
| Barn Swallow | Yes | - | - | Yes | Yes | Yes | Yes | Yes | Yes | Yes | Yes | Yes | Yes | Yes | Yes | Yes |
| Hawfinch | Yes | - | D | D | Yes | Yes | Yes | Yes | Yes | Yes | Yes | Yes | Yes | Yes | Yes | Yes |
| Gray Heron | - | - | - | M | M | - | - | - | M | - | - | - | - | - | - | M |
| Blackbird | Yes | - | - | - | Yes | Yes | Yes | Yes | Yes | Yes | Yes | Yes | Yes | Yes | Yes | Yes |

D, could not be assessed because of damages to the sections; LSH, left sinus horn only; M, macroscopic only; MRI, assessed from images from magnetic resonance imaging; Yes, macroscopic and histological assessment; - , not assessed; 1, Myocardial sinus venosus; 2i, detection of a sinus nodal structure (immunohistochemical); 2h, detection of a sinus nodal structure (histological); 3, sinoatrial valve; 4, left sinus horn; 5, Atrial trabeculations; 6, Right atrioventricular junction; 7, Pulmonary arterial valve; 8, number of pulmonary veins; 9, myocardium of pulmonary veins; 10, Dorsal ridge in left atrial antechamber; 11, Shelf between the body of the left atrium and its antechamber; 12, Left atrioventricular junction; 13, Aortic valve; 14, Main branches of the coronary arterial tree; 15, Ventral merger of atrial walls.

**Supplementary Table 2**. The cranial offset of the left atrioventricular junction compared to the right atrioventricular junction.

| Species | Offset in mm | Section (cranial) | | Section (LAVJ) | | Section (RAVJ) | | Section (caudal) | |  |
| --- | --- | --- | --- | --- | --- | --- | --- | --- | --- | --- |
| Ostrich |  | 61 | | 175-212 | | 178-242 | | 359 | |  |
| Mallard | 1.8 | 271 | | 991- | | 1171- | | 1201* | |  |
| Jackdaw | 2.43 | 122 | | 243-292 | | after 305 | | 332* | |  |
| Lesser redpoll | 1.2 | 321 | | 441-501 | | 561- | | 621* | |  |
| Collared dove | 3.3 |  | |  | |  | |  | |  |
| Common swift | 1.2 | 221 | | 561-681 | | 681- | | 821* | |  |
| Eurasian coot | 4.4 | 81 | | 361-641 | | 801- | | 821* | |  |
| Common snipe | 0.3 | 480 | | 990-1140 | | 1020- | | 1410* | |  |
| Sparrowhawk | >0,8 | 261 | | 661- | | after 741 | | 741* | |  |
| Barn owl (too damaged) | | | D | | D | | D | |  | |
| Green woodpecker | 0.8 | 701 | | 1061-1201 | | 1141- | | 1381* | |  |
| Common kestrel | 2.2 | 501 | | 881-1101 | | 1101- | | 1281* | |  |
| Budgerigar | 1.75 | 300 | | 600-725 | | 775- | | 850* | |  |
| Barn swallow | 1.2 | 120 | | 270-330 | | 390- | | 540* | |  |
| Hawfinch | >0,4 | 201 | | 601- | | after 641 | | 641* | |  |
| Blackbird | 0.6 | 691 | | 335 | | 275 | | 185* | |  |

Offset in mm, in millimeters how much cranial the first appearance of the left atrioventricular junction is to the first appearance of the right atrioventricular junction; Section (cranial), the number of the cranial-most section that contains atrial wall in it; Section (LAVJ), the sections contain left atrioventricular junction; Section (RAVJ), the sections that contain right atrioventricular junction; Section (caudal); ), the number of the caudal-most section that contains atrial wall in it; *, this section is the caudal-most section of the section series and the atrial muscle extented further caudal than this section.
